# ArchMap is a web-based platform for reference-based analysis of single-cell datasets

**DOI:** 10.1038/s41588-026-02756-y

**Published:** 2026-09-30

**Authors:** Mohammad Lotfollahi, Chelsea Bright, Ronald Skorobogat, Mohammad Moghareh Dehkordi, Xavier George, Simon Richter, Vladimir A. Shitov, Aleksandra Topalova, Helena Melcher, Noah Nussbaumer, Annika Fomm, Malte D. Luecken, Fabian J. Theis

**Affiliations:** 1 Institute of Computational Biology, Helmholtz Zentrum München, German Research Center for Environmental Health, Neuherberg, Germany; 2 Wellcome Trust Sanger Institute, Cambridge, UK; 3 Cambridge Stem Cell Institute, University of Cambridge, Cambridge, UK; 4 Institute of Lung Health and Immunity (LHI), Comprehensive Pneumology Center (CPC), Helmholtz Munich, Member of the German Center of Lung Research (DZL), Munich, Germany; 5 Department of Mathematics, Technische Universität München, Garching bei München, Germany; 6 TUM School of Life Sciences, Technical University of Munich, Freising, Germany

**Keywords:** Software, Transcriptomics

## Abstract

Leveraging single-cell reference atlases to analyze new data has brought about a paradigm shift in single-cell data science akin to the first reference genome in genomics. However, methods for performing this mapping require computational expertise and, oftentimes, considerable compute power, limiting access for researchers who may benefit the most. Here ArchMap, a no-code query-to-reference mapping tool, removes this barrier by providing all-in-one automated mapping, cell-type annotation and collaborative features to analyze single-cell datasets from a wide range of integrated, often published, reference atlases and allows the extension of atlases with the growing Human Cell Atlas and related efforts. This paves the way for a democratization of reference mapping capabilities.

## Abstract (web summary)

Existing software for mapping single-cell datasets onto reference atlases often requires coding expertise or a license. ArchMap is a free no-code query-to-reference mapping web tool compatible with Python-based mapping methods.

## Main

Large-scale integrated single-cell reference atlases have become key for understanding intercellular variation and achieving consensus on cell-type nomenclature^1–4^. A central use of these reference models has been to gain insights into new data by mapping them onto the atlas using transfer learning^5–9^, similar to using genome references in genomics^10^. However, leveraging these methods requires knowledge of programming and, nowadays, often GPU (graphics processing unit) compute; even with this know-how, setting up the complex pipelines required for atlas use is time-consuming and potentially prohibitive for users.

To address this limitation, tools that simplify the use of these methods have been developed; however, they are either limited in their focus or analysis capabilities or restricted by paywalls. For example, Azimuth^7,8^ provides a graphical user interface (GUI), allowing atlas access to its users to project new single-cell data; however, the platform lacks collaborative features, and mapping can only be performed using methods from Seurat v4. This prohibits free, no-code comparison of reference mapping methods and flexibility to choose the optimal reference mapping approach. FASTGenomics^11^ provided reference mapping to the Human Lung Cell Atlas (HLCA); however, access is no longer freely available. scGPT Hub^12^ and CellTypist^13^ are GUIs that can also be used for reference mapping and cell-type annotation, respectively; however, scGPT is a foundation model pretrained on diverse datasets and fine-tuned only on the user’s desired reference and query dataset, and thus may introduce certain biases and residual batch effects from other nonrelevant datasets. In fact, foundation models have been shown to perform worse for integration tasks compared with scVI (single cell variational inference)^14^ and are also more computationally expensive, specifically when fine-tuning. Furthermore, CellTypist on its own does not allow for the removal of batch effects, and thus, another method or tool would need to be used to integrate the query into the reference.

ArchMap (Architecture Mapping; https://www.ArchMap.bio) is a free, no-code query-to-reference mapping framework that provides compatibility with Python-based mapping methods and includes out-of-the-box cell-type label transfer, uncertainty estimation and collaborative analysis features. The fully automated approach makes query mapping accessible to users with and without coding and machine learning expertise through its intuitive GUI and access to a full code tutorial for more advanced users. A cloud-based setup enables secure sharing of results, codeless downstream analysis and collaborative annotation of the user’s mapping results. ArchMap also introduces metrics not supported in existing tools, including uncertainty quantification of label transfer that accounts for correlated latent features and a presence score quantifying the likelihood that a reference cell is present in the query. This metric was previously used to assess cell-type representation in the human neural organoid cell atlas mapped to the atlas of the developing human brain^15^.

ArchMap was developed with four guiding principles in mind: maximizing accessibility; protecting user data privacy; ensuring utility, quality and reproducibility; and enabling collaboration.

Traditional data analysis pipelines are prone to bottlenecks such as installation and package dependency issues, limiting reusability. A cloud-based setup allows for improved accessibility and convenience by removing the need for software installations and updates by the user and providing automatic access to CELL×GENE Annotate^16^ for downstream data analysis, democratizing the full analysis pipeline.

In addition, because users would typically leverage ArchMap during the early stages of data processing, when concerns about data security and privacy are highest, ArchMap is specifically designed to keep user data protected through our Google Cloud implementation. All user data are encrypted during upload and download and when stored, ensuring that only authorized parties have access at any time. Furthermore, all projects deleted by the user are automatically deleted from cloud storage after 3 days. Expert users can also run the ArchMap pipeline locally by hosting the web application themselves following our provided tutorial (Methods).

**Fig. 1:**
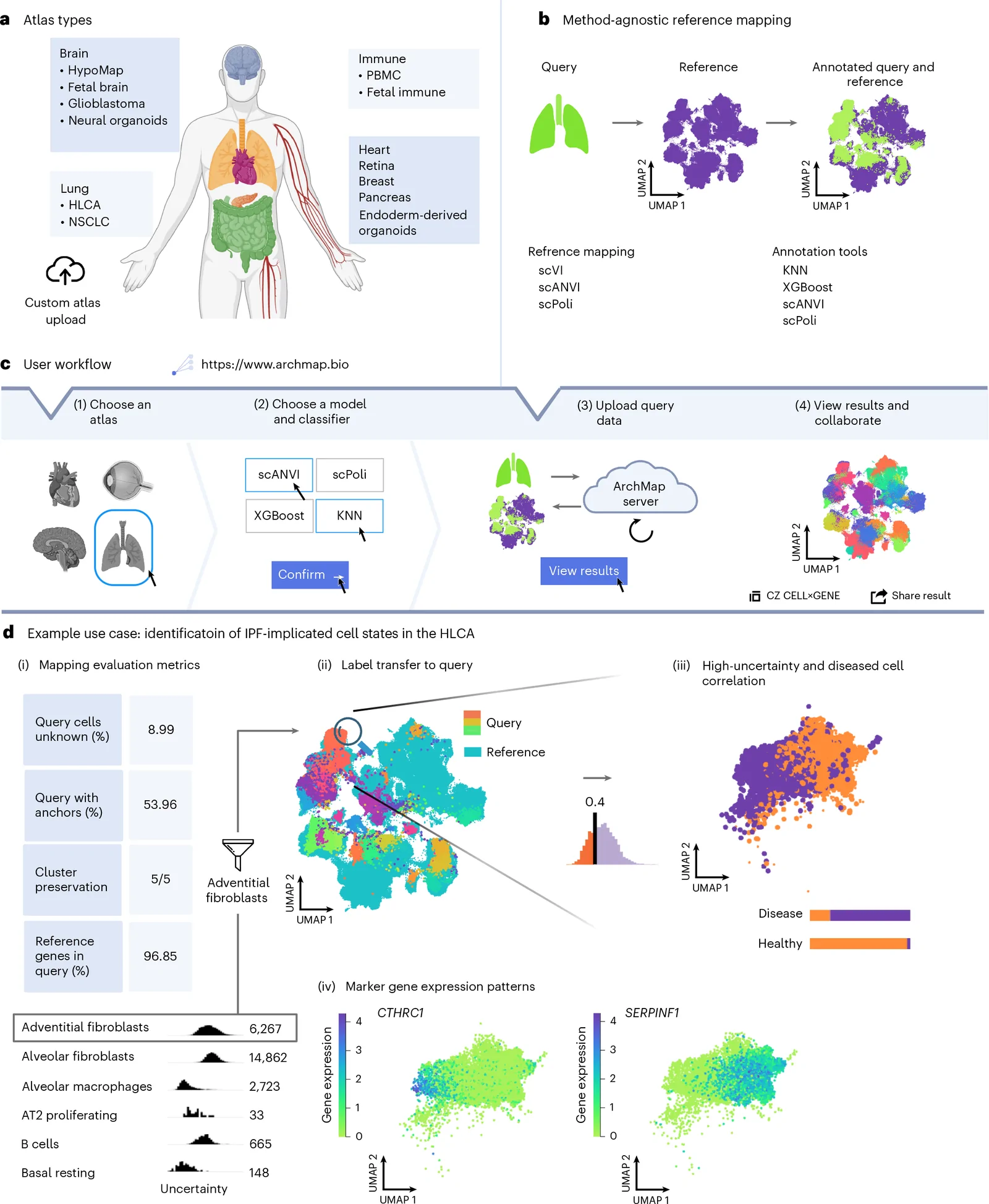
A web-based platform for automated single-cell reference mapping and annotation. **a**, Atlas types available for mapping on the ArchMap website. Atlas builders can upload their own atlases, which are published on the website after review. **b**, ArchMap’s backbone, the scArches query mapping method, enables reference mapping to atlases built with a variety of atlas learning methods. A selection of classifiers allows users to compare classification models and choose the best-performing model for their dataset based on the percentage of query cells labeled unknown. **c**, The standard ArchMap user workflow consists of atlas and model selection for mapping and cell-type label transfer; a query upload page to drag and drop or select your desired h5ad file for mapping, as well as an outline of the prerequisites the h5ad file should satisfy before upload; and finally, a results page where mappings can be visualized with a CELL×GENE plugin, where evaluation metrics can be viewed and your results can be downloaded. **d**, Use case mapping of a single-cell dataset of human samples from patients with idiopathic pulmonary fibrosis to the HLCA. (i) Post-mapping, mapping evaluation metrics can be viewed on the ArchMap website to assess mapping quality. (ii) A CELL×GENE instance can be launched to visualize the query dataset mapped onto the reference atlas. The UMAP of the mapping is colored by the cell-type label predictions for the query cells computed by ArchMap, with the reference cells grayed out to distinguish query from reference. Cell-type-specific density plots of the ArchMap-computed uncertainty scores are compared with adventitial fibroblasts shown to have a higher variability in their uncertainty scores. (iii) An association can be seen between the uncertainty scores of adventitial fibroblasts and the disease label of a cell. (iv) Marker gene expression patterns of *CTHRC1* and *SERPINF1* show higher expression in high- and low-uncertainty cells, respectively. Figure created in BioRender; Bright, C. https://biorender.com/am7ze7z (2026).

**Extended Data Fig. 1:**
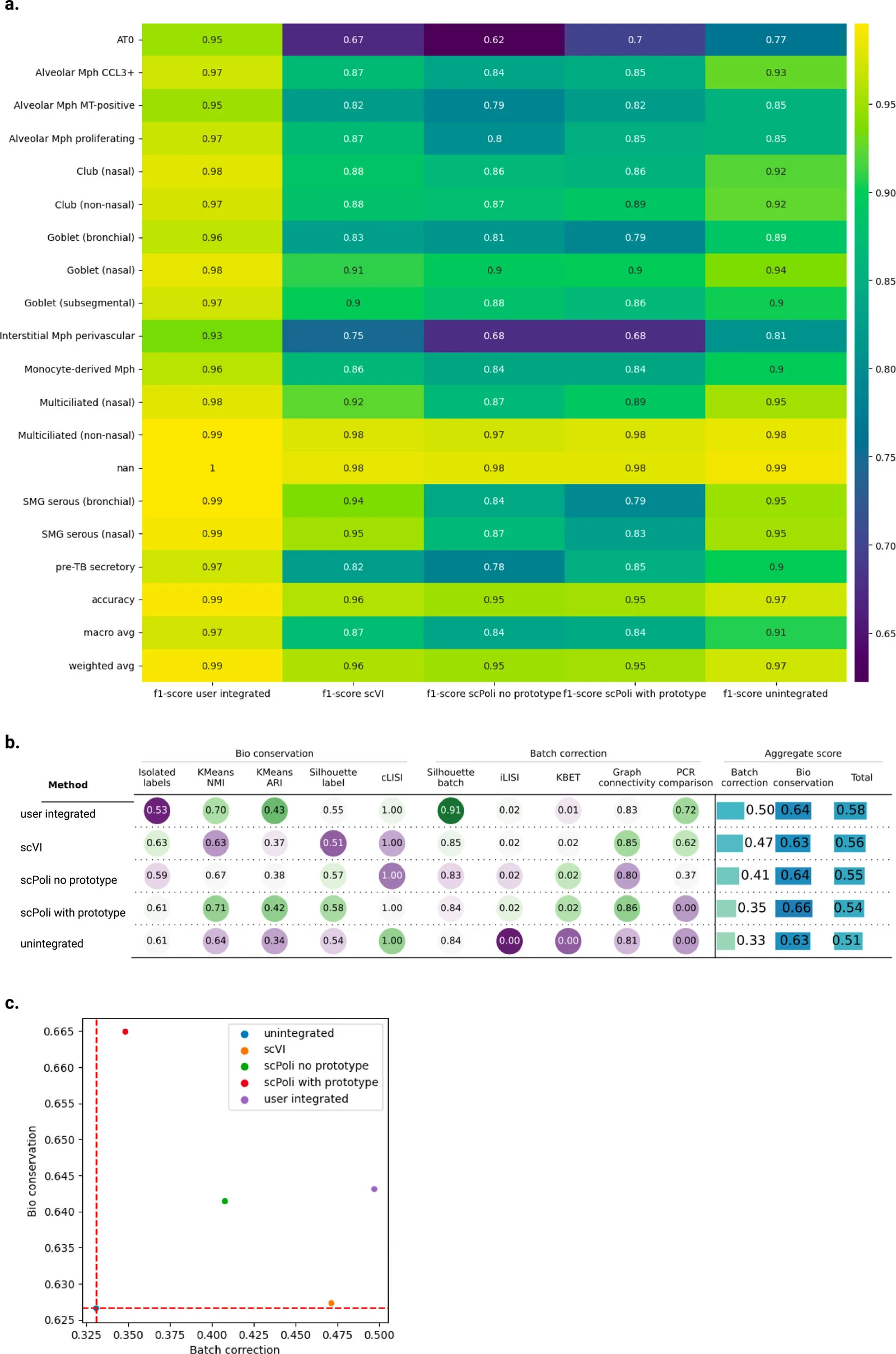
ArchMap’s atlas upload evaluation pipeline results for the HLCA. **a**, Comparison of KNN classifier F1 scores between classifiers trained on the latent representations taken from the user integrated model (scANVI), scVI, scPoli without prototype loss, scPoli with prototype loss, and PCA. Cell type labels used for ground truth were “ann_level_5” (finest annotation level) **b**, Overview of integration methods used to integrate HLCA ranked by overall score. **c**, Scatter plot comparing batch correction vs. bio conservation scores for each method. Red dashed lines represent the scores for PCA.

**Extended Data Fig. 2:**
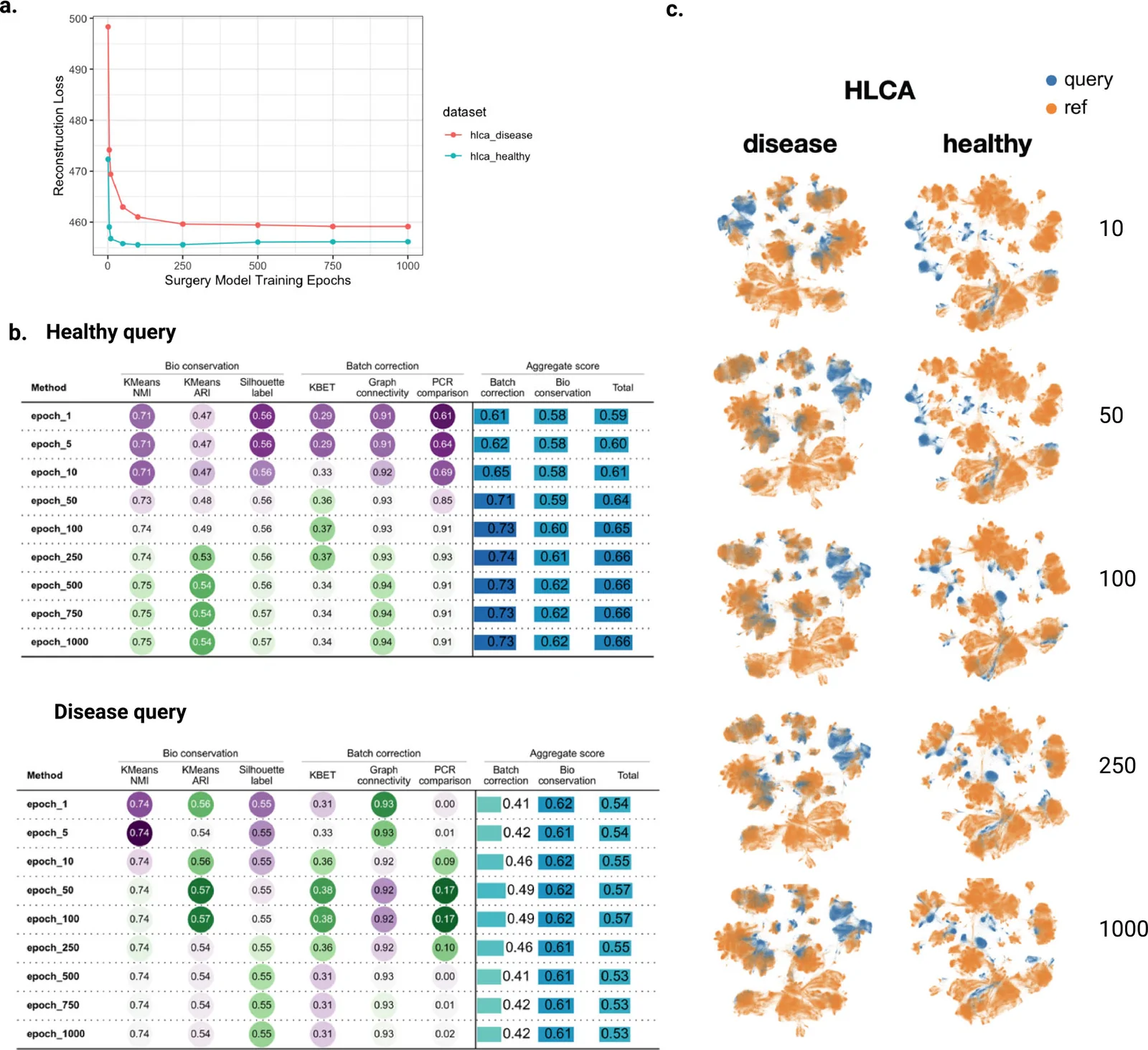
Benchmarking number of epochs used for reference mapping. Benchmark of the number of epochs to be used for query-to-reference mapping using both disease and healthy queries mapped to the HLCA. Mapping quality is evaluated using the **a**, reconstruction loss **b**, scIB metrics using the scib-metrics package, and **c**, UMAP embeddings.

**Extended Data Fig. 3:**
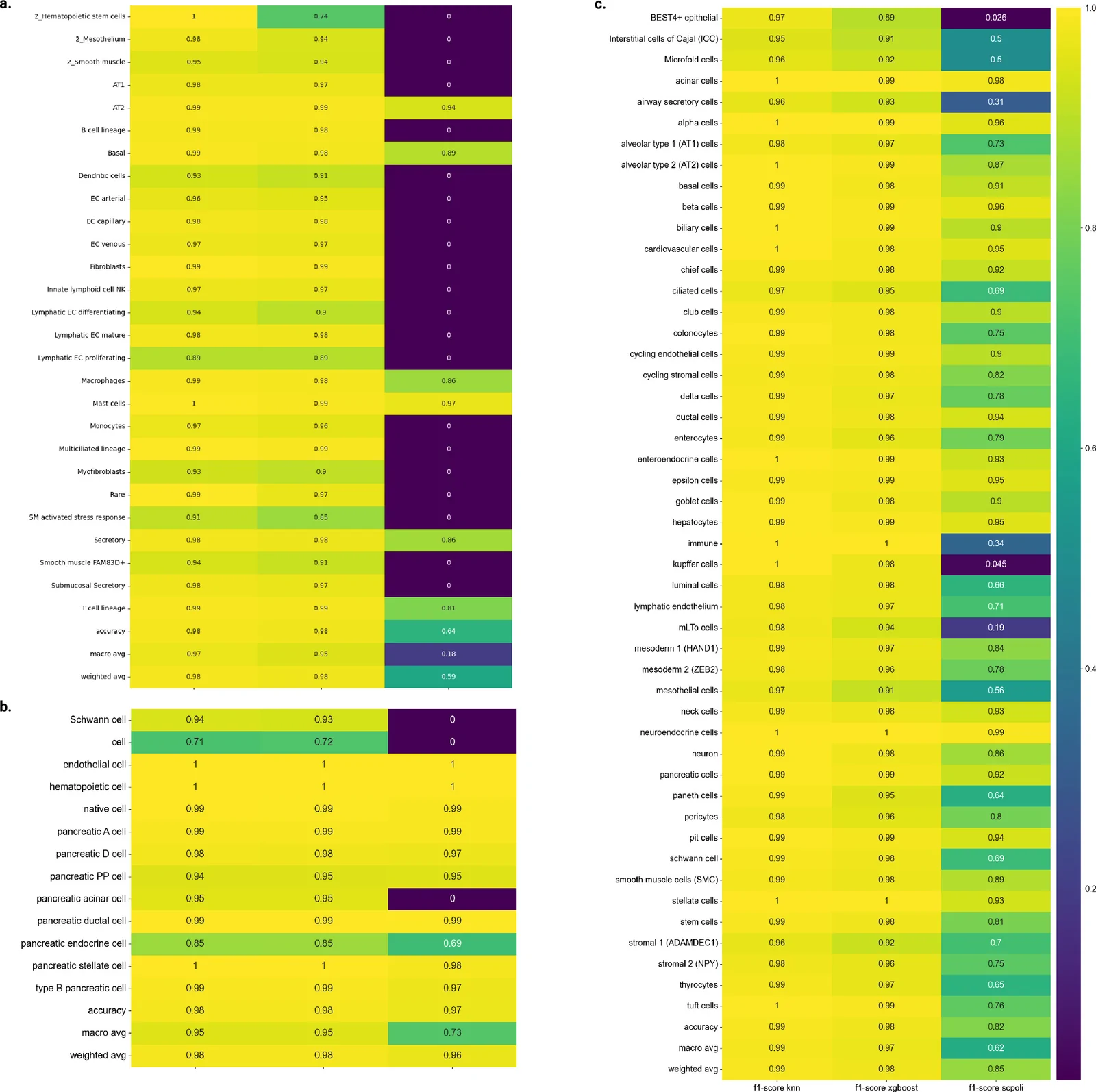
Comparing F1 scores of KNN, XGBoost, and native cell type label classifiers for pancreas, HLCA, and HNOCA. Comparison of F1 scores between KNN, XGBoost, and scANVI native classifiers for the **a**, HLCA using “ann_level_3” as ground truth and **b**, pancreas using “cell_type”. For both atlases, KNN gives the best performance. KNN also gives the best performance for finer cell type labels. **c**, Comparison of F1 scores between KNN, XGBoost, and scPoli native classifiers for HEOCA using “level_2” cell type labels as ground truth. Again KNN also gives the best performance for finer cell type labels.

To ensure the utility of ArchMap across datasets from different tissues, ArchMap is built to ingest high-quality reference atlases integrated using different methods. A wide variety of atlases are available on ArchMap, including atlases accepted by the Human Cell Atlas (HCA) as official tissue references^1,15,17–25^. In addition, scvi-hub compatibility allows for coverage of atlases with no existing preprint or publication^26^. All reference atlases available in ArchMap are the extensively evaluated, published and, where available, consortium-approved versions (for example, by the HCA^27^) of the atlases. We do not retrain models ourselves, as this voids the extensive performance evaluation and community-based annotation typically done for HCA atlases. Users can upload their own atlases for mapping or dissemination via ArchMap’s upload feature. Submitted atlases are initially marked ‘in revision’ during the integration quality checks (Methods and Extended Data Fig. 1). If they pass, atlases can be made public or kept private by the user (Fig. 1a and Methods). Mapping is made possible through scArches^28^, ArchMap’s backbone for query mapping. ScArches is a transfer learning framework that can be extended to any conditional neural-network-based data-integration method. Current compatible integration methods include scVI^6^, scANVI (single-cell annotation using variational inference)^9^ and scPoli (single-cell population level integration)^5^, with scVI and scANVI consistently being top-performing integration methods^4^. The number of training epochs for reference mapping was determined through benchmarking across datasets (Extended Data Fig. 2). Furthermore, to cover potential performance differences for user queries, the user has access to a variety of pretrained classifiers for post-mapping cell-type classification. These include ArchMap’s out-of-the-box XGBoost (extreme gradient boosting) and KNN (K-nearest neighbors) implementations, as well as scANVI and scPoli native classifiers for compatible atlases (Fig. 1b). Using 80–20 train-test splits, we assessed the performance of each classifier and found that KNN performs best across all atlases and cell-type label granularities (Extended Data Fig. 3). To assess the overall effectiveness of the mapping, ArchMap provides mapping quality metrics. Along with Euclidean uncertainty, ArchMap includes Mahalanobis uncertainty, which allows for the quantification of cell-type uncertainty where the latent features of the cell are correlated. These uncertainty metrics can be used to assess the reliability of the mapping and to identify novel cell states in query datasets. Users can relate the uncertainty scores to the transferred labels manually in CELL×GENE for all cells. A suggested cutoff of 0.5 is given in ArchMap for the uncertainty scores, where values higher than this are recommended to be labeled as unknown, as the resulting label is more uncertain than certain (Methods).

In addition, a presence score indicates which reference cell states are represented in the query dataset, helping to evaluate reference suitability (Methods).

Moreover, given the interdisciplinary nature of single-cell genomics, collaboration is crucial for effective research. ArchMap’s cloud infrastructure allows for easy and secure data sharing, as well as collaborative annotation and data analysis with CELL×GENE. To balance data sharing with privacy considerations, ArchMap users can choose to keep their data private or share them with other users within or across teams or institutions.

The ArchMap workflow involves selecting a reference atlas and classifier, uploading a query dataset for mapping and, after successful processing, exploring the mapping in CELL×GENE Annotate and downloading the results (Fig. 1c and Supplementary Note 1).

To demonstrate this workflow, we mapped a single-cell dataset of human samples from patients with idiopathic pulmonary fibrosis^29^ to the Human Lung Cell Atlas^1^ using ArchMap. Mapping quality was high, with 96.85% gene overlap and full Leiden cluster preservation (5 out of 5) (Fig. 1d and Methods). In addition, 53.96% of query cells had a corresponding reference cell (anchor) in a similar biological state, while 8.99% had high uncertainty (>0.5) and were classified as ‘unknown’. A lower percentage of anchors and higher uncertainty are expected when mapping a disease dataset to a healthy reference. Launching ArchMap’s CELL×GENE plugin, we examined transferred labels and uncertainty scores, observing notable variability in adventitial fibroblasts, a cell type associated with idiopathic pulmonary fibrosis^29^. Stratification of adventitial fibroblasts into low- and high-uncertainty groups highlighted an association between high-uncertainty scores and diseased cell labels. Differential expression analysis using CELL×GENE showed that low-uncertainty cells reflected homeostatic processes, whereas high-uncertainty cells showed fibrotic signatures, including elevated CTHRC1, a marker of fibrotic fibroblasts (Fig. 1d)^29^. These results show that ArchMap can identify disease-associated cell states and key driver genes directly within its interface, without requiring local analysis. Using uncertainty scores alone to identify novel or disease-associated cell states may not always be reliable. Residual batch effects or low-quality cells (for example, low gene counts or high mitochondrial content) can also yield high uncertainty and lead to misinterpretation. Therefore, high-uncertainty cells should be manually validated after label transfer, for example, using differential expression analysis as shown above.

**Extended Data Fig. 4:**
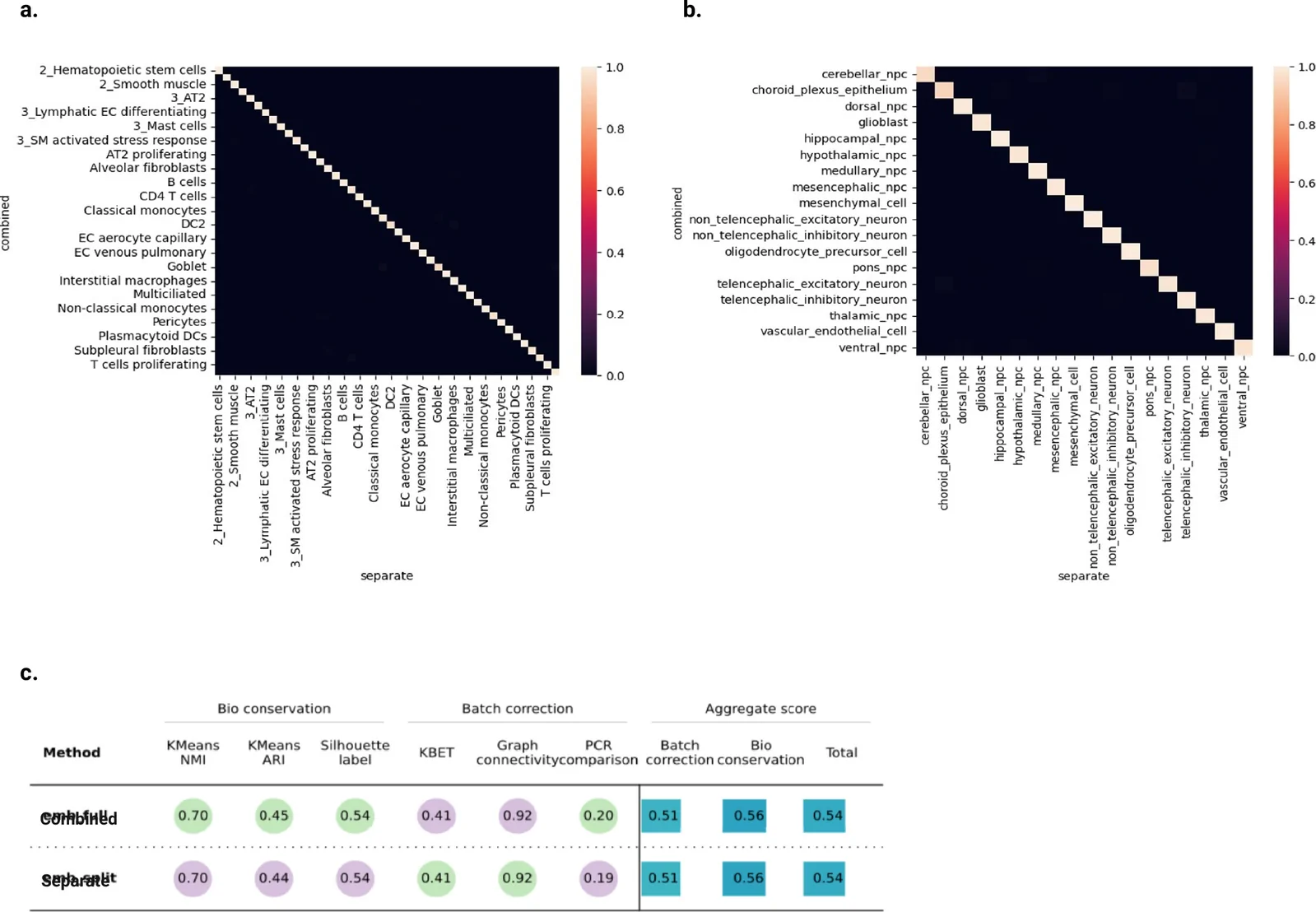
Comparing mapping and label transfer performance of separately mapped query batches vs. a single query mapping containing all batches. Comparing label transfer performance of separately mapped query batches vs. a single query mapping containing all batches for the **a**, HLCA and **b**, HNOCA. Both methods give the same results for cell type label transfer. **c**, Comparing scIB metrics between the two scenarios mapped to the HLCA.

Taken together, ArchMap enables query-to-reference mapping and out-of-the-box cell-type annotation for new data using existing references from a multitude of tissues. ArchMap automatically calculates various performance metrics, including uncertainty quantification, to evaluate mapping quality and identify novel or diseased cells. A CELL×GENE plugin allows for easy post-mapping visualization and marker gene identification. Users also have the option to share their mapping results with other users directly through ArchMap. We encourage atlas builders to promote their own work through ArchMap’s atlas upload and integration quality assessment feature. Because of server limitations, query mapping is restricted to 200,000 cells per mapping. However, users can batch their queries, submit separate projects and concatenate their mapping results easily with provided Colab notebooks (Methods). This method gives the same performance as mapping the entire query at once (Extended Data Fig. 4). With the inclusion of other modalities, such as CITE-seq (cellular indexing of transcriptomes and epitopes by sequencing) and spatial transcriptomics data, in the new generation of atlases currently being developed, we foresee ArchMap growing to accommodate multimodal reference mapping by extending to incorporate deep learning frameworks such as TotalVI (total variational inference)^30^ and MultiMIL (multimodal multiple-instance learning)^31^. Owing to the ever-increasing size of reference atlases, we also see potential in extending ArchMap to support multiple lineage atlases, which will enable users to map to a specific subset of an existing atlas. Furthermore, as existing atlases continue to be updated, ArchMap’s capability as an atlas repository can be further extended toward an open-source atlas version control platform. As it stands, ArchMap simplifies the query-to-reference mapping process for a wide variety of users and provides a more accessible alternative to traditional mapping pipelines. With its intuitive design and collaborative, secure interface, we envision ArchMap becoming a key first step for wet-lab biologists to query, annotate and understand their single-cell data.

## Methods

### Atlas preprocessing

Each atlas is preprocessed using the same steps to prepare it for use in the ArchMap machine learning pipeline. First, the atlas cells and genes are filtered to conform with the attributes of the respective trained model. Then, the atlas is minified^26^. In particular, the latent posterior parameters are calculated and stored in the .obsm attribute of the AnnData object. This step is done before any mapping to avoid computing the parameters during query mapping, thereby drastically reducing compute time. In addition, the count matrix is removed from the atlas AnnData object before mapping to further reduce compute time and the launch time of the CELL×GENE instance. The combined reference and query count matrix is then re-added to the h5ad file containing the final joint results for download.

### Query requirements

All query requirements are outlined on the query upload page under the heading ‘Query Upload Requirements!!’ when creating a mapping on the website. The query data file needs to fulfill certain requirements following upload by the user. To test whether the query matches the requirements, users can make use of our tutorials for queries with either an rds or an h5ad format. The file should be in h5ad format (rds files can be converted using the aforementioned tutorial for rds files). Furthermore, the batch or study key of the data is mandatory and should be labeled as ‘batch’. This is done by creating a new column in the observations (obs) data frame of the h5ad file. Users can make use of the Colab notebook here, which is referenced on the website and in the docs, to add the batch key to their h5ad file.

Furthermore, the size of the query has a limit of 200,000 cells owing to limitations in server capacity. If a query exceeds this limit, users can split their query into multiple batches and create separate mapping projects. The resulting h5ad files can then be concatenated post-mapping. This procedure will not affect the mapping performance (Extended Data Fig. 4); however, it is important that all cells with the same batch label remain in the same query subset following splitting. Users can use the linked Colab notebooks to separate and concatenate their query and results. These links are also referenced on the website and in the docs.

Users should also ensure that raw expression counts are saved in X of the query AnnData object, unless otherwise specified, and that either Ensembl IDs or gene names are stored in the var_names AnnData object attribute of the query. The choice of using gene symbols or Ensembl IDs in the query data is reference agnostic, and the format will be converted automatically to match the reference var_names in the ArchMap pipeline. This is done using BioMart mapping tables for the conversions of both mouse and human. To give users the opportunity to improve on this conversion in case of insufficient performance, we let them know that this conversion was done internally.

During preprocessing in the ArchMap pipeline, query genes of the uploaded query are subsetted to match the genes of the atlas, and for any missing genes, expression values are padded with zeros. This may lead to inaccuracy in the results, based on the number of missing genes in the query. The user will be made aware of this on the website during query processing. Specifically, the user will receive a message on the frontend that communicates the percentage of shared genes between the query and the reference. The user can then use this information to scrutinize the accuracy of the resulting mapping. We recommend that mappings in which more than 20% of the reference genes are missing in the query should be treated with caution (Supplementary Note 2).

### Mapping procedure

The mapping procedure in ArchMap is outlined as follows: during query upload, the batch key of the query is checked to make sure that it is labeled as ‘batch’. If not, a message is returned to the user stating that the batch key should be renamed to ‘batch’.

Next, the query data are preprocessed based on the model that was used for the atlas integration. For the case of scVI and scANVI, the prepare_query_anndata method for the respective model is run to add zero padding for missing genes in the query and to sort the genes to match the atlas. Subsequently, the setup_anndata method is used to map between the data fields used by the model and their locations in the query AnnData. These methods prepare the query to be loaded into the model using the load_query_data method implemented in the SCVI and scANVI codebase. For the case of scPoli, the preprocessing steps mentioned above for scVI and scANVI are incorporated into scPoli’s implementation of the load_query_data method.

After the query is loaded and the model is initialized with the new data, the model is trained using 100 epochs to update the reference model with the query data and obtain a joint embedding. The query latent embedding is then calculated by feeding the input query data through the updated model encoder. The query embedding is then combined with the precomputed reference embedding and stored in the .obsm AnnData attribute of the joint AnnData object under the key name ‘latent_rep’. Before combining the query with the reference, we label the query and reference cells as either ‘query’ or ‘reference’ in the ‘type’ key of the obs AnnData attribute to be used for visualization in CELL×GENE.

### Visualization and data download

CELL×GENE Annotate is used to visualize the query mapping. Users can color their mapped query by the label transfer results saved under the category on the left side that ends with ‘prediction_knn’ or ‘prediction_xgboost’ (depending on the classifier that was chosen by the user).

Before a CELL×GENE instance is created for visualization of the mapping results, the reference cells of the joint representation are subsetted to speed up computation of the UMAP (uniform manifold approximation and projection) and to guarantee conformity with CELL×GENE cell number requirements in the case of a large reference, such as the Peripheral Blood Mononuclear Cell (PBMC) atlas, which has seven million cells. For all atlases, the subsetting is done in a way that preserves the cell-type proportions in the reference data and keeps the ratio between reference and query no smaller than 5:1. The user can download the full mapping, which is in the form of a tar.gz file consisting of the combined reference and query dataset in h5ad format and the fine-tuned model; however, the neighbor graph will need to be calculated by the user for the full data if visualization is desired on the downloaded data. A Colab notebook has been prepared here, which can be used by the user to calculate the neighbor graph of their results.

### Label transfer and uncertainty quantification

The XGBoost and KNN pretrained classifiers were trained on each atlas and evaluated using an 80–20 split strategy. Comparing the F1 scores for the KNN, XGBoost and native classifiers (either scPoli or scANVI, depending on the model used to integrate the atlas), KNN performed the best for all annotation levels of all atlases.

If the pretrained classifier chosen by the user is KNN or XGBoost, the pretrained classifier and label encoding for the user-specified atlas are downloaded from the Google Cloud Platform (GCP). If the native scANVI or scPoli classifier is chosen, the respective instantiated model that has been updated on the query data is used for the classification.

ArchMap uses Euclidean and Mahalanobis uncertainty to quantify the label transfer uncertainty. The Euclidean uncertainty calculated for a cell is based on the Euclidean distance between that cell and its 15 nearest neighbors. This score is used to help the user identify novel cell states in the query data. We have added a separate column, named ‘{cell_type_key}_prediction_{classifier_name}_filtered_by_uncert>0.5’, in the output that labels cells as ‘unknown’ above a certain uncertainty threshold. Here, cell_type_key is the key in the obs attribute of the cell-type level mapped to, and classifer_name is the name of the chosen classifier (for example, KNN). The threshold is set to 0.5 and is based on the way the Euclidean uncertainty is calculated. The Euclidean uncertainty of the query cells is calculated using the weights of a weighted *k*-nearest neighbor (WKNN) classifier trained on the reference embedding, the same method used in both the original scArches^28^ and HLCA^1^ studies. The KNN classifier is applied to the query embedding, where the distances to the 15 nearest reference cells are measured for each query cell. A Gaussian weighting function is used to assign more importance to nearer reference cells and is given by the following:

$${\tilde{d}}_{ij}=\exp \left(-\frac{{d}_{ij}}{{\sigma }_{i^{\prime} }}\right),$$

where ${\tilde{d}}_{ij}$ is the Euclidean distance between the *i*th query cell and the *j*th nearest reference cell and *σ*_*i*ߣ_ = (2/*σ*_*i*_)^2^, where *σ*_*i*_ is the standard deviation of the distances to the 15 nearest reference cells.

The weight *w*_*ij*_ corresponding to the *j*th nearest reference cell of the *i*th query cell is normalized by the sum over all *K* neighbors so that the weights for each query cell sum to 1:

$${w}_{ij}=\frac{{\tilde{d}}_{ij}}{\sum _{k=1}^{K}{\tilde{d}}_{ik}}.$$

For each unique cell-type label from the reference nearest neighbors, a probability for that label is calculated by summing the corresponding reference cell weights. The reference label with the highest probability is then used as the label for the query cell. To calculate the uncertainty for that query cell, we take 1 − *p*, where *p* is the probability of the reference label used for label transfer. Thus, we set a threshold of 0.5 because a probability below 0.5 is more uncertain than certain and no label stands out as a strong prediction.

For cases in which the users are interested in considering novel cells in the query dataset, we have also included a column in the output with all transferred labels for each query cell without any threshold used. We named this column ‘{cell_type_key}_prediction_{classifer_name}’. This allows users to also obtain transferred labels for query cells with uncertainty greater than 0.5. Furthermore, the ‘{cell_type_key}_uncertainty_euclidean’ column can be used to view the exact uncertainty scores for all query cells.

The Mahalanobis uncertainty is based on the Mahalanobis distance between a query cell and the means of the Gaussian components from a Gaussian mixture model of the reference embedding, where the number of components is equal to the number of cell types. In particular, the Mahalanobis uncertainty is given by the following:

$$\frac{1}{K}\sum _{k=1}^{K}{\gamma }_{k}(x){d}_{M}(x,{\mu }_{k}),$$

where ${d}_{M}(x,{\mu }_{k})={(x-{\mu }_{k})}^{T}{\Sigma }_{\mu }^{-1}(x-{\mu }_{k})$ is the Mahalanobis distance between a query cell *x* and the mean of the *k*th Gaussian component. Here ${\varSigma }_{\mu }^{-1}$ is the inverse covariance matrix derived from the means of the *K* Gaussian components. Furthermore, ${\gamma }_{k}(x)$ is the probability of query cell *x* belonging to component *k*. This probability is the posterior probability calculated using Bayes’ theorem:

$${\gamma }_{k}(x)=P(z=k| x)=\frac{{\pi }_{k}{\mathscr{N}}(x| {\mu }_{k},{\Sigma }_{k})}{\sum _{j=1}^{K}{\pi }_{j}{\mathscr{N}}(x| {\mu }_{j},{\Sigma }_{j})},$$

where ${\mathscr{N}}(x| {\mu }_{k},{\Sigma }_{k})$ is the probability density function of the *k*th multivariate Gaussian distribution and *π*_*k*_ is the mixture weight of Gaussian component *k*.

Mahalanobis uncertainty accounts for potential correlation between the latent features of the cells. This is done by rescaling the latent features such that the covariance between any two correlated latent features is removed. If there are no correlations between the features, the distance *d*_*M*_ is equal to the Euclidean distance.

We compared the suitability of the Euclidean and Mahalanobis uncertainties in identifying disease-associated cell states in the use case presented in the main text of this paper (the mapping of an idiopathic pulmonary fibrosis dataset to the HLCA). From this analysis, we concluded that Mahalanobis uncertainty was the most appropriate to use as an uncertainty metric for this mapping (see Supplementary Note 3 for details). Based on these results, we recommend that the users make use of the Mahalanobis uncertainty to identify potential disease cell states. Although we found that the Mahalanobis uncertainty was more suitable for this use case, we have implemented both metrics in the pipeline, as the Euclidean uncertainty has been validated in the HLCA paper and may be of interest as an uncertainty metric for users based on its more established use in single-cell reference mapping.

### Performance metrics

During processing, performance metrics to quantify the quality of the mapping are calculated. These metrics include the percentage of reference genes in the query data, the percentage of cells labeled ‘unknown’, the percentage of query cells with anchors and the cluster preservation score. For the percentage of reference genes in the query data, a value less than 80% may contribute to poor mapping quality (Supplementary Note 2). The percentage of cells that are labeled as ‘unknown’ during cell-type label transfer is based on the Euclidean uncertainty score for each query cell. A query cell is classified as having an ‘unknown’ cell type if its Euclidean uncertainty score is above 0.5. A value of 0.5 is used here to take into account query datasets in which cells of the same type may look different from those of reference atlas (for example, when the query data consist of cells from diseased tissue).

The percentage of query cells with anchors represents query cells that have at least one mutual nearest neighbor among the cells of the reference dataset. These mutual nearest neighbors are termed anchors. A lower percentage of query cells with anchors may not necessarily mean poor mapping quality. A lower score may be returned for query datasets from the same tissue as the reference, but with a large proportion of cells from diseased donors. For example, mapping a dataset consisting of samples from healthy tissue^32^ to the HLCA gives a percentage of query cells with anchors of 63%. However, mapping a dataset consisting of samples from diseased tissue^29^ to the HLCA gives a percentage of query cells with anchors of 54%. We also note that the percentage of anchor cells is affected by the number of cells in the query and reference data. Thus, this metric should not be used to compare mapping quality across query or reference datasets, but only across models and classifiers. The purpose of this metric is to convey the success of the mapping and the suitability of the chosen atlas as a reference for the specific query data.

The cluster preservation score assesses how well Leiden clustering of the query dataset is preserved after the mapping process. The mean entropy of cluster labels within each neighborhood is computed for both the original and integrated query. The median of the differences in mean entropy between the original and integrated query is then calculated. Scores are scaled between 0 and 5, with 5 being the best.

### Presence score

The presence score quantifies reference cell state representation in the query dataset based on shared neighborhood structure between reference and query cells. Using a WKNN graph derived from the embedding space, we count how frequently each reference cell is identified among the *k*-nearest neighbors (*k* = 15) of query cells. This results in an initial presence score *p* for each reference cell, defined as follows:

$${p}_{i}=\sum _{q\in Q}w(q,i),$$

where *w*(*q*,*i*) is the WKNN weight of reference cell *i* in the neighborhood of query cell *q*.

To account for similarity structure within the reference dataset, the initial presence score for each reference cell is diffused using a random walk with restart performed over the reference cell transition probability matrix *T*, constructed from a reference KNN graph (*k* = 15):

$${p}^{(t+1)}=(1-\alpha )T{p}^{(t)}+\alpha {p}^{(0)},$$

with restart probability *α* = 0.1. The process is iterated for 100 rounds.

Presence scores are then log-transformed, clipped to the 1st and 99th percentiles and normalized to [0,1].

### Query mapping onto HLCA

ArchMap can be used for the identification and validation of disease-specific cell states. To show this, we mapped a single-cell dataset of human samples from healthy donors and patients with idiopathic pulmonary fibrosis to the Human Lung Cell Atlas reference and used the resulting uncertainty scores calculated on the ArchMap server to identify potential disease-associated cell states and analyze marker genes.

The percentage of reference genes in the query data is 96.85%. The percentage of cells labeled ‘unknown’ is 8.99%, and the percentage of query cells with anchors is 53.96%. The lower percentage of anchors may be a result of the query data containing a large portion of cells from diseased donors, whereas the reference only contains cells from healthy donors. The cluster preservation score is 5 out of 5, implying perfect preservation of clusters in the query.

Once the user’s query data have been mapped onto the reference using the ArchMap server, the mapping can be visualized in CELL×GENE. The embedding can be colored based on query versus reference and based on the cell-type label transfer predictions. To quantify the uncertainty of the cell-type label transfer, the Mahalanobis uncertainty is used. Coloring by this uncertainty metric, the user can identify clusters of interest that warrant further analysis. Adventitial fibroblasts can be seen as one of the cell-type clusters with the most varied uncertainty scores. We expect that clusters with both low and high uncertainty contain both healthy and diseased cells.

After filtering, we split the cluster of interest into two groups: (1) all reference cells and all query cells with uncertainty scores less than 0.4 and (2) all query cells with uncertainty scores greater than or equal to 0.4. Investigating the association between the uncertainty scores and the ground truth cell labels of healthy and diseased cells, we find a strong association between higher uncertainty scores and cells from diseased donors. This suggests that the uncertainty scores can be used as a starting point to identify disease-associated cell states (Fig. 1d).

Differential expression analysis can be performed in CELL×GENE to compare low- and high-uncertainty groups. In Fig. 1d, the embedding of adventitial fibroblasts is colored by the gene expression of *CTHRC1* and *SERPINF1*, two genes from the top 40 most differentially expressed genes for the high- and low-uncertainty groups, respectively.

### Access to scvi-hub atlases

On ArchMap, by default, users have access to the latest versions of all scANVI- and scVI-integrated scvi-hub atlases (upload of scPoli-integrated atlases is currently not available on scvi-hub). For the case where users would like to use an earlier atlas version on scvi-hub, we have included a tutorial in the ArchMap docs that walks them through downloading different versions of an atlas from scvi-hub. Users can then upload this atlas to ArchMap to perform their mapping on their desired atlas version.

Publicly available scvi-hub atlases compatible with scArches mapping include all compartments of the multiple-organ Tabula Sapiens atlas and the Human Lung Cell Atlas. These atlases are published and thus have gone through validation in the form of peer review. However, for atlases uploaded to scvi-hub in the future, we will make available on ArchMap only those with a preprint, publication or model evaluation results in the model card in scvi-hub, as described here. Users who would like to map their data to a scvi-hub atlas that does not meet these requirements can upload the desired atlas and run ArchMap’s benchmarking process as described here.

### Atlas upload revision

To support community uploads of reference atlases, a platform user must first be an admin. To become an admin, the user must reach out to ArchMap’s development team. This can be done by checking the box ‘Request for Beta feature’ after registration to ArchMap. Once approved, the user can start contributing by providing additional atlases. A user-uploaded atlas must first go through a revision process before it is publicly available on the website. The revision process consists of the following evaluation steps: (1) benchmarking the user-uploaded model used for integration with scVI, scPoli with prototype learning, scPoli without prototype learning and principal component analysis (PCA; no batch effect removal). Benchmarking is done using scIB metrics^4^. (2) Comparing the KNN classifier F1 scores between classifiers trained on the latent representations taken from the user-integrated model, scVI, scPoli with prototype learning, scPoli without prototype learning and PCA (see documentation for full details of the benchmarking process). Once the atlas has been successfully benchmarked, the user will be able to download the benchmarking report from the website and can send this report to the ArchMap team at ArchMap.bio@gmail.com. This report contains only the benchmarking evaluation as described in points (1) and (2) above. Thus, no private data are shared through email. Once the report has been successfully reviewed to ensure integration quality, the atlas uploader can choose whether to make their atlas available to the public for mapping or to keep their atlas private.

### Framework architecture

ArchMap’s backend is written in Express.js and runs on a Node.js server. The machine learning pipeline is a stand-alone service, primarily based on the scArches package for reference mapping. The pipeline is written in Python.

The backend and the machine learning pipeline are hosted on GCP. The backend primarily runs on Google App Engine. Google Cloud Storage is used to store persistent data, such as project mapping results and annotation files. The machine learning pipeline and ephemeral CELL×GENE visualization instances are run on Google Cloud Run.

#### Software tools for analysis

All analyses were done using Python version 3.10. For query-to-reference mapping and downstream analysis, the following packages with their corresponding versions were used: scArches (source installation using Git+ (https://github.com/theislab/scarches.git@speed_improvement_merge)), Scanpy (version 1.9.1), XGboost (version 1.7.4), scikit-learn (version 1.5.2), scvi-tools (version 1.2.0) and scIB metrics (version 0.5.0). A full list of all package versions that are used in the ArchMap website mapping pipeline are available at https://github.com/theislab/archmap_data/blob/mappingjob/mapping/scarches_api/requirements.txt.

### Running ArchMap locally

We have added detailed information on how users can install ArchMap locally on our GitHub. Expected costs for hosting the web application amount to 200 euros a month for multiple mappings per day for Google Cloud services (including storage and compute). For users who are interested only in the mapping pipeline or who are not familiar with Node.js, we have also provided a tutorial in the form of a Python notebook. This tutorial provides users with the exact mapping pipeline that is used in ArchMap but can be run locally. Users only need to create an environment by running the provided bash script and then running the notebook. By default, the tutorial is set to map a provided query to the HLCA. If users would like to use their own data and a different reference, they can download the desired reference from ArchMap and change the input and output paths in the tutorial notebook.

### Number of epochs for mapping

For all query-to-reference mappings, a fixed value of 100 epochs is used for fine-tuning the reference model. This number was chosen by running mappings of both healthy and disease query data, with approximately 70,000 and 118,000 cells, respectively, to the HLCA and increasing the number of epochs from 1 to 1,000. We evaluated the mappings using the reconstruction loss, scIB metrics and UMAP embeddings (Extended Data Fig. 2). We found that reconstruction loss for both query datasets plateaus after approximately 100 epochs (Extended Data Fig. 2a). Furthermore, for the healthy query, we see all scIB metrics continue to increase up until approximately 250 epochs, at which point the kBET (k-nearest neighbour batch effect test) and PCR (principal component regression) comparison decrease slightly. After this decrease, at approximately 500 epochs, they then remain constant, along with all other metrics. For the disease query, we see that past 100 epochs, PCR comparison, kBET, and KMeans ARI (adjusted Rand index) begin to decrease. Thus, for all mappings on ArchMap, we have set the value for the number of epochs to 100, because for the majority of scIB metrics, we see no further improvement past this number (Extended Data Fig. 2b). Furthermore, for both the reconstruction loss and the UMAP embeddings, we see no change or improvement in integration when training for more than 100 epochs (Extended Data Fig. 2c).

### Number of overlapping genes between query and reference

During mapping, only genes used to build the reference atlas model can be used to map a query dataset to this atlas, requiring the genes of query datasets to be subsetted to match the atlas gene set. To better understand the effect of this subsetting on query-to-reference mapping, we benchmarked query mapping quality under varying overlap of profiled genes in the HLCA reference (Supplementary Note 2). This experiment has allowed us to provide a more informative guideline to users mapping their query to a reference atlas on ArchMap. Based on our benchmark, we recommend that the overlap of genes between the query and reference should be no lower than 80%.

### Reporting summary

Further information on research design is available in the Nature Portfolio Reporting Summary linked to this article.

## Online content

Any methods, additional references, Nature Portfolio reporting summaries, source data, extended data, supplementary information, acknowledgements, peer review information; details of author contributions and competing interests; and statements of data and code availability are available at https://doi.org/10.1038/s41588-026-02756-y.

## Supplementary information

Supplementary Information Supplementary Notes 1–3 and Figs. 1–3.

Reporting Summary

Peer Review File

## Data Availability

All data, including all atlases and data used to showcase the ArchMap use case, are public. All atlases are referenced in the paper and can be downloaded directly from the ArchMap website at https://www.archmap.bio/#/references/atlases. The original, published dataset that was used for the ArchMap use case in this paper is available at Gene Expression Omnibus under accession number GSE132771.

## Code availability

The machine learning pipeline for mapping query data to reference atlases and implementing all downstream computation tasks is available via GitHub at https://github.com/theislab/archmap_data/tree/mappingjob. The code for the frontend and backend implementation of ArchMap is available via GitHub at https://github.com/theislab/archmap/tree/mapjob. The source code of both repositories is available via Zenodo at https://doi.org/10.5281/zenodo.21908600 (ref. ^33^). The ArchMap website is available at https://www.ArchMap.bio.

## References

[1] Sikkema, L. et al. An integrated cell atlas of the lung in health and disease. *Nat. Med.* **29**, 1563–1577 (2023). 10.1038/s41591-023-02327-2 PMC10287567 37291214

[2] Regev, A. Science forum: the Human Cell Atlas. *Elife* 10.7554/eLife.27041 (2017). PMC5762154 29206104

[3] HuBMAPConsortium The human body at cellular resolution: the NIH Human Biomolecular Atlas Program. *Nature* **574** 192–187 (2019). 10.1038/s41586-019-1629-x PMC6800388 31597973

[4] Luecken, M. D. et al. Benchmarking atlas-level data integration in single-cell genomics. *Nat. Methods* **19**, 41–50 (2022). 10.1038/s41592-021-01336-8 PMC8748196 34949812

[5] De Donno, C. et al. Population-level integration of single-cell datasets enables multi-scale analysis across samples. *Nat. Methods* **20**, 1683–1692 (2023). 10.1038/s41592-023-02035-2 PMC10630133 37813989

[6] Lopez, R., Regier, J., Cole, M. B., Jordan, M. I. & Yosef, N. Deep generative modeling for single-cell transcriptomics. *Nat. Methods* **15**, 1053–1058 (2018). 10.1038/s41592-018-0229-2 PMC6289068 30504886

[7] Hao, Y. et al. Dictionary learning for integrative, multimodal and scalable single-cell analysis. *Nat. Biotechnol.* **42**, 293–304 (2023). 10.1038/s41587-023-01767-y PMC10928517 37231261

[8] Hao, Y. et al. Integrated analysis of multimodal single-cell data. *Cell* **184**, 3573–3587.e29 (2021). 10.1016/j.cell.2021.04.048 PMC8238499 34062119

[9] Xu, C. et al. Probabilistic harmonization and annotation of single-cell transcriptomics data with deep generative models. *Mol. Syst. Biol.* **17**, e9620 (2021). 10.15252/msb.20209620 PMC7829634 33491336

[10] Lotfollahi, M., Yuhan Hao, Theis, F. J. & Satija, R. The future of rapid and automated single-cell data analysis using reference mapping. *Cell* **187**, 2343–2358 (2024). 10.1016/j.cell.2024.03.009 PMC11184658 38729109

[11] Scholz, C. J. et al. FASTGenomics: an analytical ecosystem for single-cell RNA sequencing data. Preprint at *bioRxiv* 10.1101/272476 (2018).

[12] Cui, H. et al. scGPT: toward building a foundation model for single-cell multi-omics using generative AI. *Nat. Methods* **21**, 1470–1480 (2024). 10.1038/s41592-024-02201-0 38409223

[13] Domínguez Conde, C. et al. Cross-tissue immune cell analysis reveals tissue-specific features in humans. *Science* **376**, eabl5197 (2022). 10.1126/science.abl5197 PMC7612735 35549406

[14] Kedzierska, K. Z., Crawford, L., Amini, A. P. & Lu, A. X. Zero-shot evaluation reveals limitations of single-cell foundation models. *Genome Biol.* **26**, 101 (2025). 10.1186/s13059-025-03574-x PMC12007350 40251685

[15] He, Z. et al. An integrated transcriptomic cell atlas of human neural organoids. *Nature* **635**, 690–698 10.1038/s41586-024-08172-8 (2024). PMC11578878 39567792

[16] Megill, C. et al. cellxgene: a performant, scalable exploration platform for high dimensional sparse matrices. Preprint at *bioRxiv* 10.1101/2021.04.05.438318 (2021).

[17] Salcher, S. et al. High-resolution single-cell atlas reveals diversity and plasticity of tissue-resident neutrophils in non-small cell lung cancer. *Cancer Cell* **40**, 1503–1520.e8 (2022). 10.1016/j.ccell.2022.10.008 PMC9767679 36368318

[18] Braun, E. et al. Comprehensive cell atlas of the first-trimester developing human brain. *Science* **382**, eadf1226 10.1126/science.adf1226 (2023). 37824650

[19] Litviňuková, M. et al. Cells of the adult human heart. *Nature* **588**, 466–472 (2020). 10.1038/s41586-020-2797-4 PMC7681775 32971526

[20] Ruiz-Moreno, C. et al. Harmonized single-cell landscape, intercellular crosstalk and tumor architecture of glioblastoma. Preprint at *bioRxiv* 10.1101/2022.08.27.505439 (2022).

[21] Steuernagel, L. et al. HypoMap—a unified single-cell gene expression atlas of the murine hypothalamus. *Nat. Metab.* **4**, 1402–1419 (2022). 10.1038/s42255-022-00657-y PMC9584816 36266547

[22] Suo, C. et al. Mapping the developing human immune system across organs. *Science* **376**, eabo0510 (2022). 10.1126/science.abo0510 PMC7612819 35549310

[23] Xu, Q. et al. An integrated transcriptomic cell atlas of human endoderm-derived organoids. *Nat. Genet.* **57**, 1201–1212 10.1038/s41588-025-02182-6 (2025). PMC12081310 40355592

[24] Reed, A. D. et al. A single-cell atlas enables mapping of homeostatic cellular shifts in the adult human breast. *Nat. Genet.* **56**, 652–662 (2024). 10.1038/s41588-024-01688-9 PMC11018528 38548988

[25] Chen, R. et al. Integrated multi-omics single cell atlas of the human retina. Preprint at *Research Square*10.21203/rs.3.rs-3471275/v1 (2023).

[26] Ergen, C. et al. Scvi-hub: an actionable repository for model-driven single cell analysis. Preprint at *bioRxiv* 10.1101/2024.03.01.582887 (2024). PMC12446071 40921843

[27] Rozenblatt-Rosen, O., Stubbington, M. J. T., Regev, A., & Teichmann, S. A. The Human Cell Atlas: from vision to reality. *Nature*10.1038/550451a (2017). 29072289

[28] Lotfollahi, M. et al. Mapping single-cell data to reference atlases by transfer learning. *Nat. Biotechnol.* **40**, 121–130 (2021). 10.1038/s41587-021-01001-7 PMC8763644 34462589

[29] Tsukui, T. et al. Collagen-producing lung cell atlas identifies multiple subsets with distinct localization and relevance to fibrosis. *Nat. Commun.* **11**, 1920 (2020). 10.1038/s41467-020-15647-5 PMC7174390 32317643

[30] Gayoso, A. et al. Joint probabilistic modeling of single-cell multi-omic data with totalVI. *Nat. Methods* **18**, 272–282 (2021). 10.1038/s41592-020-01050-x PMC7954949 33589839

[31] Litinetskaya, A. et al. Multimodal weakly supervised learning to identify disease-specific changes in single-cell atlases. Preprint at *bioRxiv* 10.1101/2024.07.29.605625 (2024).

[32] Delorey, T. M. et al. COVID-19 tissue atlases reveal SARS-CoV-2 pathology and cellular targets. *Nature* **595**, 107–113 (2021). 10.1038/s41586-021-03570-8 PMC8919505 33915569

[33] Bright, C. et al. ArchMap. *Zenodo*10.5281/zenodo.21908600 (2026).

