# Supplementary material for: ArchMap is a web-based platform for reference-based analysis of single-cell datasets
Source: Nat Genet. 2026 Sep 30;58(10):2469–73. doi: 10.1038/s41588-026-02756-y (PMC13649598; doi:10.1038/s41588-026-02756-y)
Supplement: Supplementary file 1 [file 41588_2026_2756_MOESM1_ESM.pdf]

nature genetics

Supplementary information https://doi.org/10.1038/s41588-026-02756-y

ArchMap is a web-based platform for reference-based analysis of single-cell datasets

In the format provided by the authors and unedited

Supplementary Notes

Supplementary Note 1

The ArchMap user workflow starts with choosing a reference atlas and classifier for query mapping and label transfer, respectively. Upon atlas and model selection, the user is provided the necessary instructions to upload their dataset to be queried against the chosen reference model. Once the query dataset is uploaded, the user can track the processing status of each of their mapping projects in the custom “Your Mappings” page. Here, users can also view the mapping quality metrics automatically calculated by ArchMap post mapping. Once processing is completed, a CellxGene Annotate instance can be launched with the click of a button to display the joint embedding of reference and query data. In this visualisation, users can display uncertainty and presence scores calculated for each cell and make use of all CellxGene Annotate features including adding categorical cell annotations and conducting differential gene expression analysis. Users are able to download an h5ad file containing the combined reference and query data with transferred annotations and the fine-tuned model used for mapping.

Supplementary Note 2

During mapping, only genes used to build the reference atlas model can be used to map a query dataset to this atlas. As such, query dataset genes are subsetted to match the atlas gene set and zeros are added whenever an atlas gene is not profiled in the query dataset. It has been shown that a lower overlap of profiled genes between query and reference datasets may lead to poor mapping results[23]. To better understand this limitation of query to reference mapping, we benchmarked query mapping quality under varying overlap of profiled genes in the HLCA reference. We harmonized cell type labels for a new dataset, and used 3 different methods to select genes to drop to test reference mapping. These 3 methods represent an omission of random genes, a best case scenario (least informative genes are dropped), and a worst case scenario (most informative genes are dropped). To identify least and most informative genes for our query dataset (Meyer_2021_5prime dataset from HLCA Extended), we trained an XGBoost model on the count data for cell type classification. For our three separate approaches, we mapped the subsetted query datasets to the HLCA to see at which percentage overlap between the reference and query we start to see a degradation in performance. Reference mapping was evaluated by visualizing the resulting embedding space (UMAP) and testing label transfer performance (F-1 score).

From our results we see that for both methods of taking the most informative genes and a random sample, 50% overlap already allows for clustering of cells to distinct areas on the reference embedding (Supplementary Figure 1 a,c). Subsetting to only the most informative genes, however, is not a realistic scenario as mapping with a 50% overlap between query and reference does not guarantee that all genes are informative of the cell types in the query. Furthermore, compared with taking a random sample, considering only genes that are least informative of cell type provides a lower bound to mapping performance. Thus, in order to estimate this lower bound, we focus from here on the results from our “worst case scenario” approach. From Supplementary Figure 1 b and d, we see that most cell types begin to separate towards a specific cluster in the reference from a 70% overlap between

reference and query. Furthermore, there is a minimal difference in the UMAP embedding between 90% and 100% overlap. Supplementary Figure 2 shows the corresponding F-1 scores for each cell type obtained by classifying the query cells using a KNN classifier trained on the reference embedding with the harmonised annotation level ann_level_3 used as the cell type labels. We see that for an overlap of 50%, we can still obtain good classification for cell types such as Fibroblasts and AT2 that are likely well represented by the chosen genes and for which we have a larger amount of training data. However, for most cell types, we have poor classification performance. We see a noticeable improvement in classification performance for almost all cell types as the percentage overlap increases; however, for a number of cell types, the performance remains poor and is due to the underrepresentation of these cell types in the reference.

We conclude from this benchmarking analysis that the number of overlapping genes between the query and reference is an important metric to use and a gene overlap of less than 80% will likely be the cause of poor quality cell type annotations, specifically for cell types that are not well-represented by the overlapping genes. Based on our benchmark, we have updated our guideline to recommend that the overlap of genes between the query and reference should be no lower than 80%. At this level we expect that for 80% of the cell types, the F-1 score will fall within 15% of the optimal performance (Supplementary Figure 2). However, since results likely vary with the query and reference used, it is important that the user also takes into account the provided presence score and uncertainty scores as well as the other mapping quality metrics provided in ArchMap to better assess mapping quality.

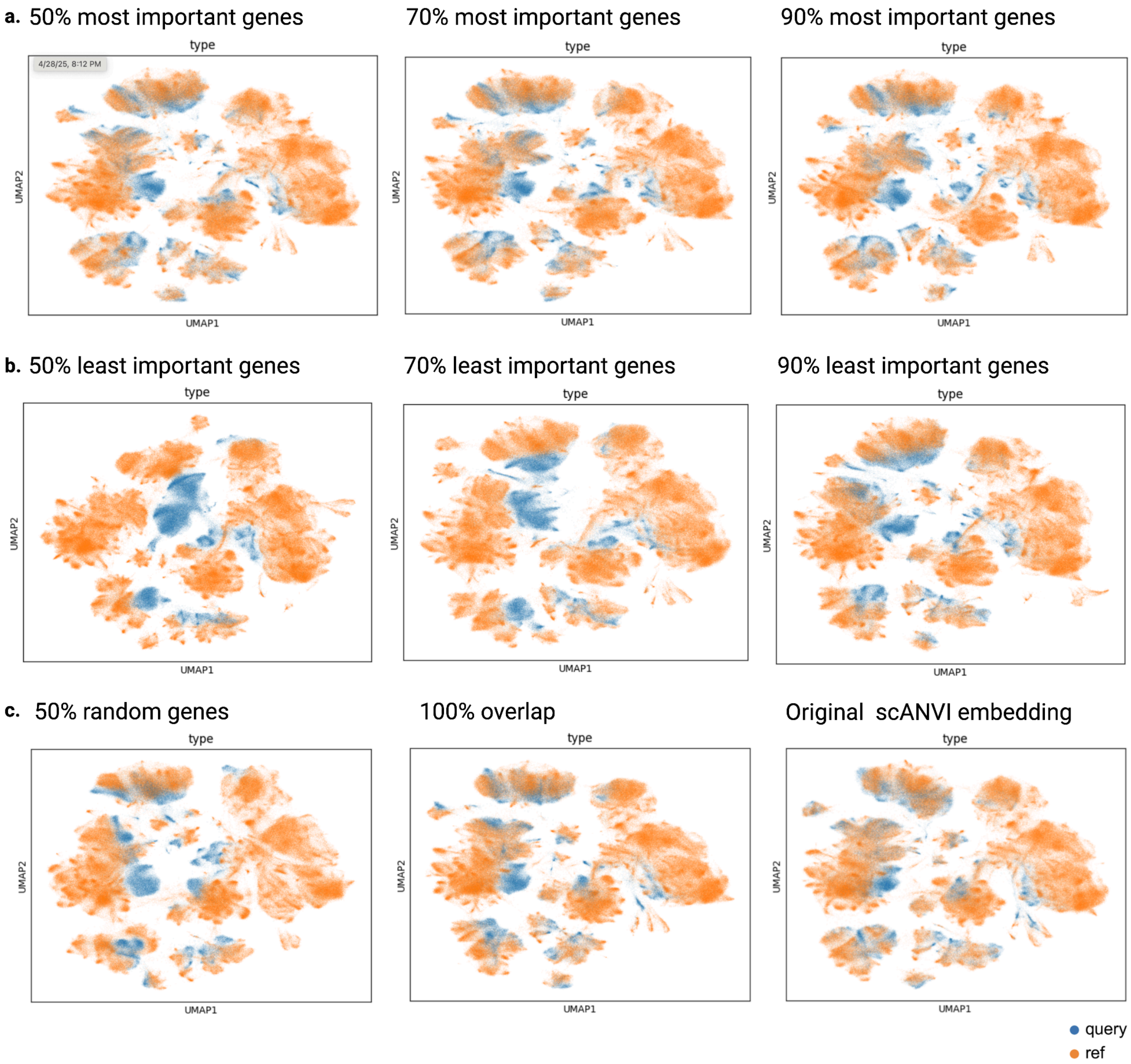

Supplementary Figure 1: Benchmarking the number of overlapping genes between reference and query

Benchmark of the number of overlapping genes between the HLCA reference and a healthy query with harmonized cell type labels taken from the HLCA extended atlas. The subset of overlapping genes was chosen using 3 different methods: **a.** removing least informative genes (best case scenario), **b.** removing most important genes (worst case scenario), and **c.** an omission of random genes. Gene importance scores were obtained from an XGBoost classifier trained on the query count data for cell type prediction. **d.** UMAP showing the mapping results using 100% overlap (left) and UMAP showing the embedding from the HLCA extended atlas subsetted to the HLCA core and query data used in this benchmark (right) (reference n=584900 cells; query n=104712 cells)

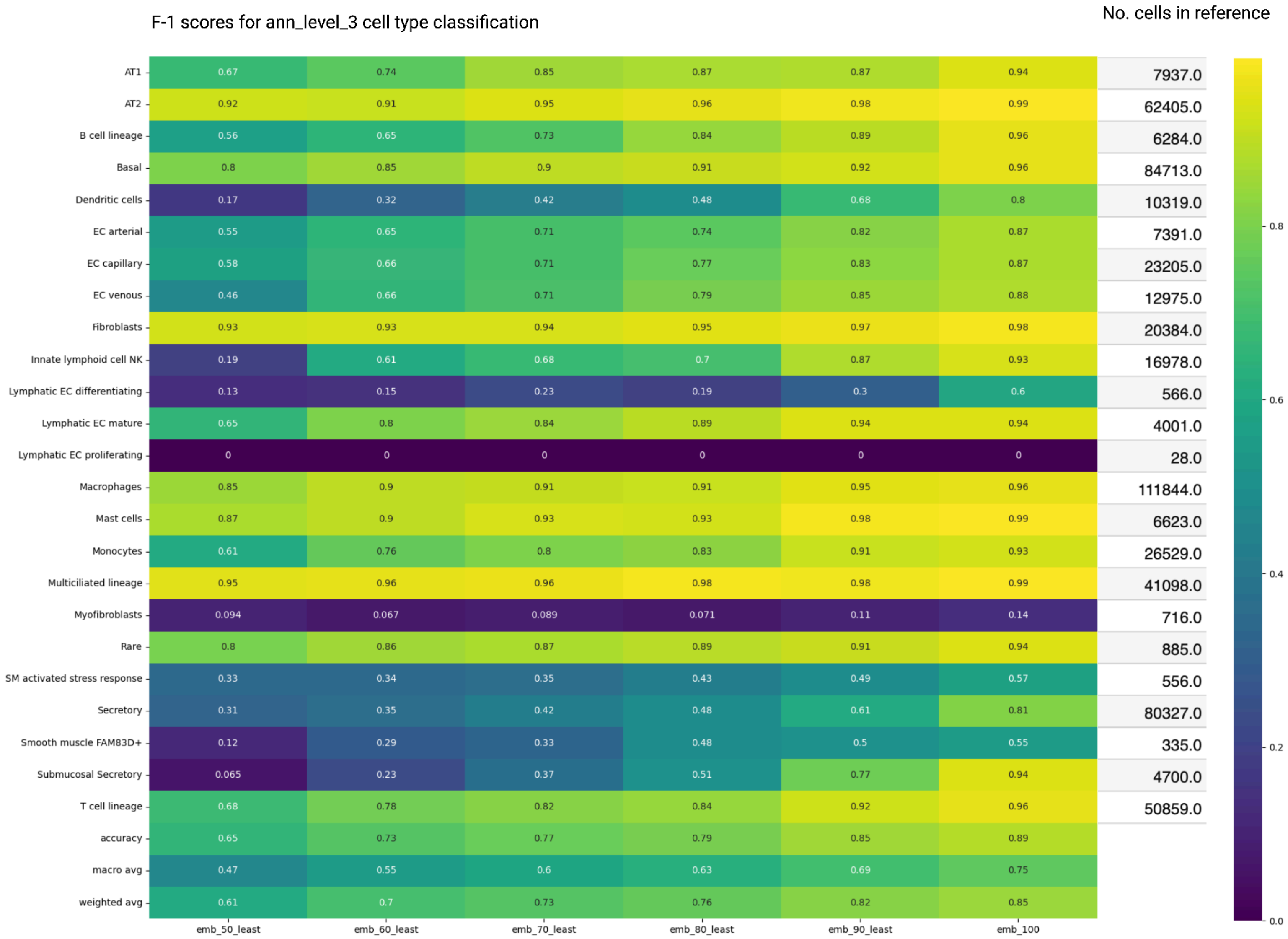

Supplementary Figure 2: Evaluating cell type classification F-1 scores for worst case scenario (keeping least important genes for mapping)

Benchmark of the number of overlapping genes between the HLCA reference and a healthy query with harmonized cell type labels taken from the HLCA extended atlas. F-1 scores evaluate cell type classification performance using a KNN classifier trained on the reference embedding. Genes were chosen based on the worst case scenario (least informative genes for classifying cell type) to obtain a lower bound on a threshold of the number of overlapping genes needed for mapping (reference n=584900 cells; query n=104712 cells).

Supplementary Note 3

Two uncertainty metrics have been incorporated into ArchMap to assess the uncertainty of the mapping of a query dataset to a reference atlas. Both estimates assign a score to each cell in the mapped query dataset to quantify the confidence of the cell type label transferred from the reference to the query cell. A detailed explanation of these two estimates is given in Methods (Label transfer and uncertainty quantification). The Euclidean uncertainty estimate has been used previously to quantify the uncertainty of cell type label transfer in the context of query to reference mapping. This estimate was introduced in the scArches manuscript and validated in the HLCA manuscript. The Mahalanobis uncertainty is not an established uncertainty metric in the context of single-cell reference mapping; however, it is formulated to account for the covariance structure in the reference data used for transferring labels, preventing the estimated distance between reference and query cells from becoming artificially large due to correlated latent features. This is important for single-cell data as gene expression patterns are highly correlated. Thus we conducted the following comparison to understand which uncertainty estimate is more appropriate for our specific use case, that is, the mapping of a single-cell dataset of human samples from healthy donors and patients with IPF to the HLCA.

We focus on a specific cluster, namely adventitial fibroblasts in the IPF dataset (also focused on in the use case in the manuscript). Adventitial fibroblasts have a high variation across cells in both uncertainty scores and is a cell type known to be associated with IPF. We see for this cluster that the Spearman correlation is 0.47 (p-value: 0.0), indicating a moderate positive correlation between the uncertainty estimates. To obtain a better understanding of which uncertainty estimate is more representative of the underlying disease variation in the data, we look at the relationship between each uncertainty score given to the cells and their disease label. In particular, we look at how the percentage overlap between “high” uncertainty cells and “diseased” cells compare between the two uncertainty metrics as we vary the threshold that is used to classify a “high” vs “low” uncertainty cell (Supplementary Figure 3a). For the Mahalanobis uncertainty, we see that for uncertainty thresholds between 0.2 and 0.3, all cells classified as having “high” uncertainty are cells from a diseased patient in the query dataset. This is, however, not the case for the Euclidean uncertainty, which has a percentage overlap of just below 80% for an uncertainty threshold of 0.2 and decreases further to just above 60% for a threshold of 0.3. For both uncertainty metrics, we see that the percentage overlap with “ground truth” diseased cells decreases as we increase the threshold further towards 1. This makes sense as we are increasing the number of cells that we classify as “normal” or “healthy”, as we increase the threshold.

We also conducted differential gene expression analysis (DGEA) for (1) the ground truth cell labels of healthy vs diseased, and (2) high vs low uncertainty groups for different thresholds for both uncertainty scores. We then compared the differentially expressed genes (DEGs) obtained for both uncertainty scores in (2) to the list obtained in (1) for the 200, 50, and 20 top DEGs (Supplementary Figure 3b). We see that for the Mahalanobis uncertainty and a threshold between 0.3 and 0.4, the overlap is the highest and remains between 70 and 80 percent when using 200, 50, and 20 DEGs for comparison. This communicates that the number of overlapping genes is high for both high and lower ranked DEGs. We also see that this stability in the number of DEGs compared was maintained for the Euclidean uncertainty and for other thresholds. We also see, for the Mahalanobis uncertainty, that the percentage

overlap drops significantly below an uncertainty threshold of 0.3 and above that of 0.4. This may be because when the threshold is below 0.3, we have more cells classified as “high” uncertainty/“diseased” cells and thus we may have more genes that are not disease-specific that are included in the top DEG list, replacing disease-specific genes. On the other hand, when the threshold is above 0.4, we have less cells classified as “high” uncertainty/“diseased” cells and thus there is a higher likelihood of not including disease-associated genes in the top DEG list. The Euclidean uncertainty does not have the same variation that we see for the Mahalanobis uncertainty and the percentage overlap remains below 60%, with the percentage gradually decreasing with a higher uncertainty threshold (Supplementary Figure 3b).

From these results, we concluded that the Mahalanobis uncertainty was most appropriate to use as an uncertainty metric for this mapping, based on the use case of the metric as an estimate for the disease association of a cell. This is because the Mahalanobis uncertainty performed better than the Euclidean uncertainty at representing the disease-associated cell states by a high uncertainty score when the threshold was set below 0.55. For a threshold above this, both metrics performed poorly. Additionally, for thresholds between 0.25 and 0.45, the high uncertainty cells assigned using the Mahalanobis uncertainty had DEGs that were more aligned with the DEGs of the “ground truth” disease cell states. Lastly, we note that a threshold of 0.4 was chosen to compare the association between the uncertainty scores and the ground truth cell labels of healthy and diseased in the main text of the paper based on this analysis as it represents an upper bound on the thresholds that would be appropriate to identify diseased cell states.

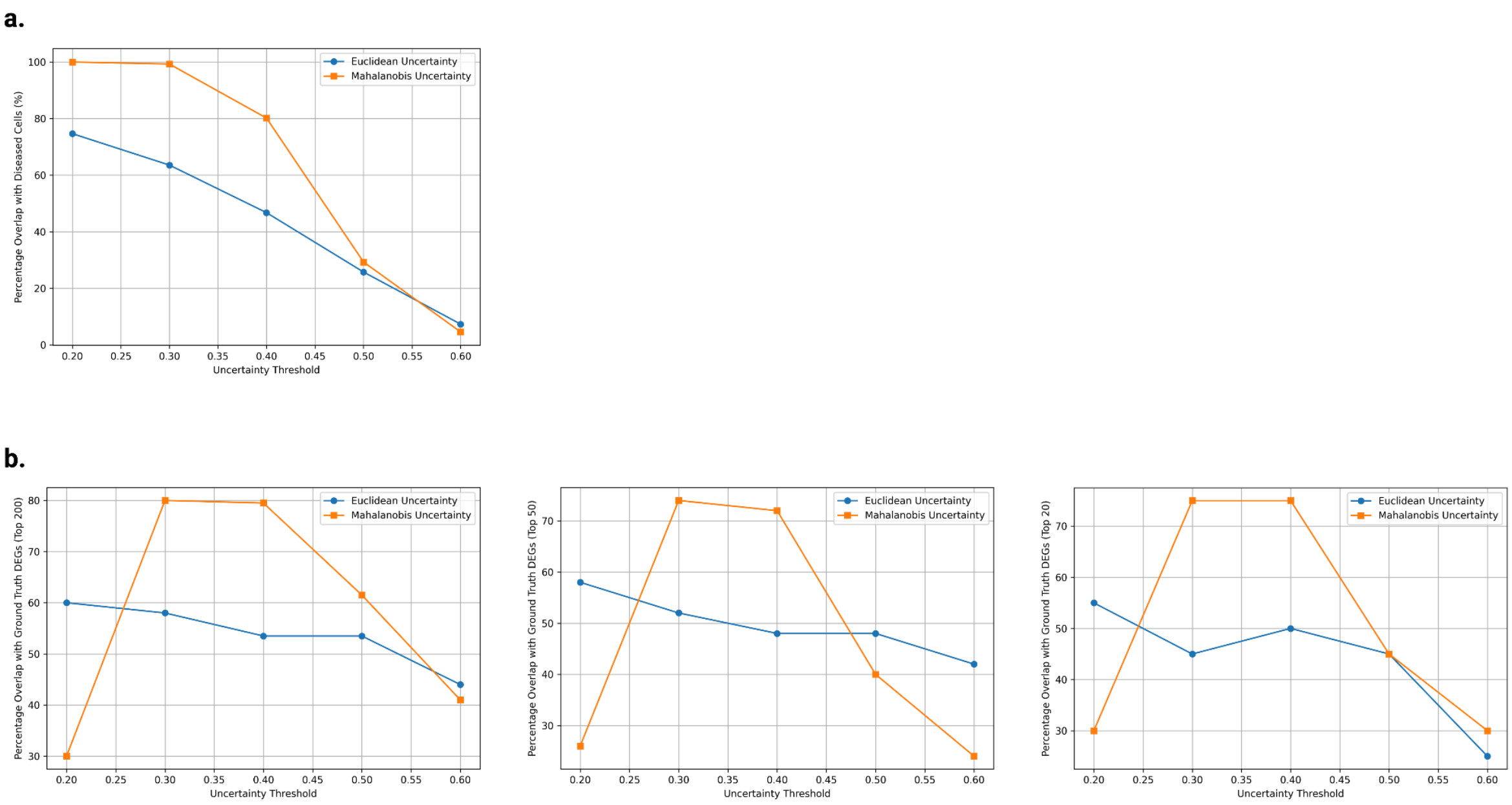

Supplementary Figure 3: Comparing Mahalanobis and Euclidean cell type label uncertainty scores for the mapping of an IPF query dataset to the HLCA. a. The percentage overlap between “high” uncertainty cells and “diseased” cells for the two uncertainty estimates as we vary the threshold that is used to classify a “high” vs “low” uncertainty cell. **b.** Percentage overlap between the DEGs obtained for high vs low

uncertainty groups for different thresholds for both uncertainty scores and those of “ground truth” healthy vs diseased cells (reference n=584900 cells; query n=80020 cells).
