# Supplementary material for: ArchMap is a web-based platform for reference-based analysis of single-cell datasets
Source: Nat Genet. 2026 Sep 30;58(10):2469–73. doi: 10.1038/s41588-026-02756-y (PMC13649598; doi:10.1038/s41588-026-02756-y)
Supplement: Supplementary file 3 [file 41588_2026_2756_MOESM3_ESM.pdf]

nature portfolio

Peer Review File

ArchMap: A web-based platform for reference-based analysis of single-cell datasets

Corresponding Author: Professor Fabian Theis

This file contains all editorial decision letters in order by version, followed by all author rebuttals in order by version.

Attachments originally included by the reviewers as part of their assessment can be found at the end of this file.

Version 0:

Decision Letter:

18th Oct 2024

Dear Fabian,

Your Brief Communication, "ArchMap: A web-based platform for reference-based analysis of single-cell datasets" has now been seen by 3 referees. You will see from their comments below that while they find your work of interest, some important points are raised. We are interested in the possibility of publishing your study in Nature Genetics, but would like to consider your response to these concerns in the form of a revised manuscript before we make a final decision on publication.

Briefly, the three reports strike a notably similar tone and arrive at the same overall judgement: ArchMap could be a useful tool for the field but it is currently falling short in (1) charaterisation and benchmarking and (2) usability. We note that many of the specific comments raised by the referees appear repeatedly, suggesting these are indeed major issues!

Thankfully, plentiful guidance is also provided to address these issues and, to our reading, none of the requests are unreasonable. We would, unsurprisingly, particularly emphasise the questions on usability as particularly important: if ArchMap is to be broadly adopted by the field it must be user-friendly, especially for non-bioinformatics-expert users.

To guide the scope of the revisions, the editors discuss the referee reports in detail within the team, including with the chief editor, with a view to identifying key priorities that should be addressed in revision and sometimes overruling referee requests that are deemed beyond the scope of the current study. We hope that you will find the prioritized set of referee points to be useful when revising your study. Please do not hesitate to get in touch if you would like to discuss these issues further.

We therefore invite you to revise your manuscript taking into account all reviewer and editor comments. Please highlight all changes in the manuscript text file. At this stage we will need you to upload a copy of the manuscript in MS Word .docx or similar editable format.

We are committed to providing a fair and constructive peer-review process. Do not hesitate to contact us if there are specific requests from the reviewers that you believe are technically impossible or unlikely to yield a meaningful outcome.

When revising your manuscript:

*1) Include a “Response to referees” document detailing, point-by-point, how you addressed each referee comment. If no action was taken to address a point, you must provide a compelling argument. This response will be sent back to the referees along with the revised manuscript.

*2) If you have not done so already please begin to revise your manuscript so that it conforms to our Brief Communication format instructions, available
here.
Refer also to any guidelines provided in this letter.

*3) Include a revised version of any required Reporting Summary: https://www.nature.com/documents/nr-reporting-summary.pdf
It will be available to referees (and, potentially, statisticians) to aid in their evaluation if the manuscript goes back for peer review.
A revised checklist is essential for re-review of the paper.

Please be aware of our guidelines on digital image standards.

Please use the link below to submit your revised manuscript and related files:

Link Redacted

Note: This URL links to your confidential home page and associated information about manuscripts you may have submitted, or that you are reviewing for us. If you wish to forward this email to co-authors, please delete the link to your homepage.

Please do not hesitate to contact me if you have any questions or would like to discuss these revisions further.

Nature Genetics is committed to improving transparency in authorship. As part of our efforts in this direction, we are now requesting that all authors identified as ‘corresponding author’ on published papers create and link their Open Researcher and Contributor Identifier (ORCID) with their account on the Manuscript Tracking System (MTS), prior to acceptance. ORCID helps the scientific community achieve unambiguous attribution of all scholarly contributions. You can create and link your ORCID from the home page of the MTS by clicking on ‘Modify my Springer Nature account’. For more information please visit please visit www.springernature.com/orcid.

We look forward to seeing the revised manuscript and thank you for the opportunity to review your work.

Sincerely,

Michael Fletcher, PhD
Senior Editor, Nature Genetics
ORCiD: 0000-0003-1589-7087

Referee expertise: all have experience in genomics/bioinformatics, including specifically single-cell analysis.

Reviewers' Comments:

Reviewer #1 (Remarks to the Author):

In this manuscript, the authors describe ArchMap, a web tool for mapping single-cell query data to a collection of reference atlases using one of several previously published mapping models and classification methods. The user uploads their data to a private location, but can choose to share the results with other users. The aim is that the tool should be usable by scientists without requiring them to write complex code.

Overall I found the manuscript well written, the website appealing and the documentation of the tool quite extensive. Below are my specific comments based on reading the manuscript and trying the tool with an example data set.

1. I found the introductory paragraphs (lines 31-81) a bit hard to follow and somewhat lacking linear structure. The same statements are made several times (e.g., that coding limits usability for non-computational users), and properties of ArchMap are described (line 46) before it is properly introduced (line 55). I think this section could be made easier to read by making some rearrangements of the text.

2. In the introduction, it is noted that for Azimuth, 'mapping can only be done using methods from Seuratv4. This prohibits compatibility to atlases integrated using strictly python methods'. Later, ArchMap is described as providing a framework that 'extends to python-based mapping methods'. To me, this indicates that also the previous R/Seurat-based methods should be supported (hence the 'extends'). However, as far as I can understand this is not the case, and ArchMap rather provides an alternative, for specific python-based methods. I think this should be made clearer.

I tested the tool with the 'pbmc3k' dataset provided in the 'TENxPBMCData' R package. More precisely, I obtained and converted this dataset to h5ad format as follows (R v4.4.1, Bioconductor 3.19):

```
> x <- TENxPBMCData::TENxPBMCData("pbmc3k")
> x$batch <- x$Individual
```

```
> colData(x) <- colData(x)[, c("Sample", "Barcode", "batch")]
> zellkonverter::writeH5AD(x, file = "pbmc3k.h5ad", X_name = "counts")
```
After this, opening the object in python I can confirm that x.var_names are indeed human Ensembl IDs.
```
>>> x.var_names
Index(['ENSG00000243485', 'ENSG00000237613', 'ENSG00000186092',
...
```
I then attempted to upload this object to test it with the human PBMC atlas, the scPoli model and the KNN classifier.

3. For the atlas and the model selection, hovering over an option shows me the 'select' option, but even after clicking on it, it does not change to 'deselect' (it still says 'select'), as opposed to the classifier selection (which now says 'deselect'). I was initially unsure whether I had in fact selected the atlas/model.

4. After uploading the data set, the processing fails with an error "zero-dimensional arrays can not be concatenated". It is not clear to me where this comes from, and if it indicates an issue with the uploaded data. Due to this error, I was not able to explore the tool further.

5. It would be very helpful to have guidance on how to properly prepare the input data starting from a SingleCellExperiment or Seurat object - e.g. in terms of which checks should be performed before saving the object as an h5ad file. Having a way to validate the h5ad file locally before uploading would also be helpful (alternatively, that the object would be validated before the processing starts and that any deviations from the expectations would be clearly stated).

6. After failing to upload my own data set, I wanted to see if there was a demo data set provided with the application. However, this does not seem to be the case currently (there is a 'Select Demo Dataset' header, but it says that there are no existing datasets available). I think this would be helpful to provide as well, for users to be able to explore the platform.

7. Currently the tool requires the user to upload their data to the Google Cloud. Despite security assurances, I imagine it may still deter some users or organizations. I was just wondering if, with the advances of WebAssembly tools, the authors envision a way that the computations could be performed in the user's browser at some point.

8. The paper mentions 'access to full code tutorials for more advanced users'. Could you expand on this statement? Does this refer to tutorials describing how to perform the whole process locally (this would be cool)? I was not able to find a reference to such tutorials. The notebooks that are referenced cover things like addition of the batch variable and separating/concatenating large data sets, which would be required from all users to make their dataset compatible with the ArchMap assumptions.

9. Generally, 'ensemble IDs' should be 'Ensembl IDs' (in the manuscript and on the webpage)

10. Line 396: upun -> upon

Reviewer #2 (Remarks to the Author):

The authors present ArchMap a web-platform for atlas integration and cell-type label transfer. This is an important contribution to democratize access to single-cell reference models by providing advanced reference mapping functions to a wider user base.

General comments:
Currently, I wouldn’t recommend using ArchMap due to technical issues. I am convinced that the authors can overcome these technical issues.
To highlight these issues. Despite several attempts, I couldn’t overcome warnings about CSC sparse format and process datasets downloaded from CELLXGENE. The error message is not helpful as it doesn’t highlight whether query or reference data is the issue here. For a web platform, I expect the developers to perform some conversions to reduce preprocessing effort. I was finally successful mapping a part of the HLCA data (see provided reviewer login for error messages).
I uploaded a new trained scVI model together with data without any count data (minified format from scvi-hub). The atlas was uploaded but I couldn’t see any report (scIB metrics or similar) and after logging out the atlas seems publicly available (marked as under revision). Allowing the atlas uploader a first look at the quality metrics would circumvent this. The quality metrics (scIB metrics etc.) should be accessible to the uploader.
For all scvi-hub models, no classifier can be selected and even scANVI doesn’t support classification. I wasn’t able to test these models as training led to the error ‘Native’.

In addition, I have the following concerns.

Privacy concerns
The authors highlight data privacy for uploaded query datasets and provide a solid solution. However, it seems that all reference atlases are hosted publicly. Is there an option to have private reference atlases? Is the only option to self-host the full ArchMap webpage?
The developers provide guidance on setting up a custom ArchMap web interface on their GitHub README. Could they argue about monthly costs and time to set up an interface?

Model training
The author benchmark splitting datasets into separate batches highlighting preservation of cell-type annotation. This is a very coarse measurement especially as small deviations from identity are not well-displayed using a confusion matrix. The authors should compare reconstruction loss or neighborhood preservation in latent space between both scenarios.
The authors provide interpretation for various quality scores. However, I am not aware that these scores and especially cut-offs are well established in the literature. The authors should benchmark this aspect. E.g. the authors argue that if 20% of the genes are missing (depending on the location in the documentation 15%) the results should be further scrutinized. Instead of giving a fixed value, another approach could be to subset the reference data to the overlapping genes and benchmarking performance on a corrupted small subset of the reference dataset. This won't add much computational cost but would give better guarantees.
The authors argue about multi-modal data (including ATAC) and have an API for totalVI models in GitHub. Is totalVI supported in ArchMap? The limitation to 50k cells will likely be stricter on ATAC data due to dataset size. How many cells can likely be mapped for multi-ome data?
I found that even for 500k cells scArches was initialized but never finished. An error should be raised for large datasets.
The authors state that depending on the atlas ensemble gene IDs or gene symbols are expected. The conversion between those should optimally happen on-the-go within ArchMap to reduce computational burden.
The authors provide KNN and XGBoost as classifiers and argue that if the uncertainty is high XGBoost should be used. How do they reach this statement?
scANVI is benchmarked on the published HLCA model. However, their developers raised an issue with the classifier and fixed the model more recently: https://docs.scvi-tools.org/en/stable/tutorials/notebooks/scrna/scanvi_fix.html. Benchmarking should be performed after retraining on the same dataset for scANVI and scPoli in extended figure 2.
For Euclidean and Mahalanobis distances as well as Presence Score the calculation doesn't become fully clear (value of 0.5 as cut-off doesn't make sense with the description). The authors should provide a formula or pseudocode.
In the anchor computation, the ratio of Anchor Cells is likely highly affected by the number of cells in query and reference data. Computation should be performed on a fixed number of reference and query cells.
The authors train models for 10 epochs. This is much lower than in tutorials for scArches. The number of epochs should be benchmarked (on datasets with less than 50k cells).
An additional field in the CELLXGENE session should be created in which all cells (query and reference have cell-type labels). Currently only query cells are labeled.

Reproducibility using ArchMap:
After successful mapping, fine-tuned models should be accessible via download and not only the dataset.
All reference atlases should be version-controlled. Scvi-hub models e.g. provide different versions that can't be accessed on ArchMap. Uploading reference atlases to scvi-hub could be a solution to allow reproducibility and easy access to reference models (huggingface provides version control). AWS or GitHub would be other options here.

Minor:
Scvi-hub models are supported in ArchMap. However, when navigating to https://www.archmap.bio/#/references/atlases these don't appear but are only visible when creating a mapping: https://www.archmap.bio/#/genemapper/create. This will create confusion for users that want to access a specific model stored in scvi-hub.
In the list of available tools for reference mapping the authors should refer to https://www.celltypist.org/ for Celltype annotation and scGPT hub for reference embedding using a foundational model. I think using specific reference models and query embedding provides advanced use cases beyond these two platforms.
Figure 1D IV doesn't have axis labels.
The authors argue that scvi-hub provides access to unpublished models. Is it a requirement for ArchMap to have a preprint? What type of validation is performed on scvi-hub models? Are they less trustworthy in ArchMap?
Presence score: My understanding is that it measures which reference cell states are present in the query dataset. Could the authors clarify this and rephrase lines 141-143.
When discussing minification, the authors should cite scvi-hub to demonstrate why this is a reasonable step.
Line 277: In the GitHub API the developers use load_query_anndata of scVI and scANVI.
Line 300: It is unclear to me to which Cell Number Requirement the authors are referring? Cellxgene is compatible with millions of cells.

Reviewer #2 (Remarks on code availability):

No issues with the published code. Navigation is easy and I can follow aspects of the package. See above for detailed questions about the code.

Reviewer #3 (Remarks to the Author):

Please see uploaded file

Version 1:

Decision Letter:

Our ref: NG-BC66689R

27th Mar 2026

Dear Dr. Theis,

Thank you for submitting your revised manuscript "ArchMap: A web-based platform for reference-based analysis of single-cell datasets" (NG-BC66689R). It has now been seen by the original referees and their comments are below. The reviewers find that the paper has improved in revision, and therefore we'll be happy in principle to publish it in Nature Genetics, pending minor revisions to satisfy the referees' final requests and to comply with our editorial and formatting guidelines.

If the current version of your manuscript is in a PDF format, please email us a copy of the file in an editable format (Microsoft Word or LaTex)-- we can not proceed with PDFs at this stage.

We are now performing detailed checks on your paper and will send you a checklist detailing our editorial and formatting requirements soon. Please do not upload the final materials and make any revisions until you receive this additional information from us.

Thank you again for your interest in Nature Genetics Please do not hesitate to contact me if you have any questions.

Sincerely,

Margot Brandt, PhD
Senior Editor
Nature Genetics
https://orcid.org/0000-0002-9434-794X

Reviewer #1 (Remarks to the Author):

In the revised submission, the authors have nicely addressed my original comments. I was able to test the webtool and it worked as advertised. I only have a few remaining comments/suggestions on this front:

1. I think it would be useful to have an idea of the size of the tar.gz file that will be downloaded before starting the download. In addition, I could imagine that some users would appreciate being able to download a simple text file with just the query cells and the annotations produced by the tool (which would be much smaller than the current tar.gz file, and also easier to integrate with existing workflows and processed data objects).

2. What is the difference between reference datasets with Modalities "RNA-seq" and "rna"?

3. The code in the 'CheckFormatsRds.ipynb' notebook does not execute properly (it fails at Bioconductor installation) since the latest colab runtime version provides R v4.5.2, while Bioconductor version 3.20 (which is hardcoded in the notebook) requires R v4.4. I would suggest to decide on a strategy here - either fix the R version to an old one (which may work for conversion as long as the AnnData format does not change) or don't fix the Bioconductor version (and make sure that the code still runs properly with newer Bioconductor releases). In general (see also the code comments below), this may point to a broader issue - how do the authors ensure that code distributed via notebooks is regularly tested and kept up-to-date and functional?

Reviewer #1 (Remarks on code availability):

1. I attempted to follow the instructions from https://archmap-docu.readthedocs.io/en/latest/tutorials/map_local.html, including downloading the example data from the Google Drive and running the tutorial from https://github.com/theislab/archmap_data/blob/mappingjob/mapping/tutorial.ipynb, but it failed with the following error:

```
>>> from scarches_api.utils.utils import replace_X_on_disk, check_h5ad_format
Traceback (most recent call last):
File "<stdin>", line 1, in <module>
ImportError: cannot import name 'check_h5ad_format' from 'scarches_api.utils.utils'
(archmap_data/mapping/scarches_api/utils/utils.py)
```

I have scarches version 0.6.1

2. The code in the tutorial could be cleaned up - for example, some modules (scanpy, pandas) are imported several times

Reviewer #2 (Remarks to the Author):

The reviewers have significantly improved the manuscript as well as the web portal. In my opinion this article now mertis publication in Nature Genetics. Especially the benchmarks provided for training speed (number of epochs) as well as missing genes will benefit the community beyond ArchMap.
I would like the authors to address that the provided distance metric for detection of new cell-types might not be robust in all

scenarios and that manual verification remains key after automatic cell-type transfer. For example, if a new cell-type is relatively close to an existing cell-type such as monocytes compared to macrophages, these cells would not be flagged but the annotation is still inaccurate.

Reviewer #3 (Remarks to the Author):

The authors have addressed all of my concerns, and the website worked very nicely in our tests.

Open Access This Peer Review File is licensed under a Creative Commons Attribution 4.0 International License, which permits use, sharing, adaptation, distribution and reproduction in any medium or format, as long as you give appropriate credit to the original author(s) and the source, provide a link to the Creative Commons license, and indicate if changes were made.
In cases where reviewers are anonymous, credit should be given to 'Anonymous Referee' and the source.
The images or other third party material in this Peer Review File are included in the article's Creative Commons license, unless indicated otherwise in a credit line to the material. If material is not included in the article's Creative Commons license and your intended use is not permitted by statutory regulation or exceeds the permitted use, you will need to obtain permission directly from the copyright holder.
To view a copy of this license, visit https://creativecommons.org/licenses/by/4.0/

Editor’s comments:

Dear Fabian,

Your Brief Communication, "ArchMap: A web-based platform for reference-based analysis of single-cell datasets" has now been seen by 3 referees. You will see from their comments below that while they find your work of interest, some important points are raised. We are interested in the possibility of publishing your study in Nature Genetics, but would like to consider your response to these concerns in the form of a revised manuscript before we make a final decision on publication.

Briefly, the three reports strike a notably similar tone and arrive at the same overall judgement: ArchMap could be a useful tool for the field but it is currently falling short in (1) characterisation and benchmarking and (2) usability. We note that many of the specific comments raised by the referees appear repeatedly, suggesting these are indeed major issues!

Thankfully, plentiful guidance is also provided to address these issues and, to our reading, none of the requests are unreasonable. We would, unsurprisingly, particularly emphasise the questions on usability as particularly important: if ArchMap is to be broadly adopted by the field it must be user-friendly, especially for non-bioinformatics-expert users.

To guide the scope of the revisions, the editors discuss the referee reports in detail within the team, including with the chief editor, with a view to identifying key priorities that should be addressed in revision and sometimes overruling referee requests that are deemed beyond the scope of the current study. We hope that you will find the prioritized set of referee points to be useful when revising your study. Please do not hesitate to get in touch if you would like to discuss these issues further.

We therefore invite you to revise your manuscript taking into account all reviewer and editor comments. Please highlight all changes in the manuscript text file. At this stage we will need you to upload a copy of the manuscript in MS Word .docx or similar editable format.

We are committed to providing a fair and constructive peer-review process. Do not hesitate to contact us if there are specific requests from the reviewers that you believe are technically impossible or unlikely to yield a meaningful outcome.

When revising your manuscript:

*1) Include a “Response to referees” document detailing, point-by-point, how you addressed each referee comment. If no action was taken to address a point, you must provide a compelling argument. This response will be sent back to the referees along with the revised manuscript.

*2) If you have not done so already please begin to revise your manuscript so that it conforms to our Brief Communication format instructions, available here [nature.com].
Refer also to any guidelines provided in this letter.

*3) Include a revised version of any required Reporting Summary:
https://www.nature.com/documents/nr-reporting-summary.pdf [nature.com]
It will be available to referees (and, potentially, statisticians) to aid in their evaluation if the manuscript goes back for peer review.
A revised checklist is essential for re-review of the paper.

Dear Michael,

Thank you for your comments and the reviewer comments and the opportunity to adapt our manuscript to address them, particularly focusing on benchmarking and usability. We have now used this feedback, adapted our manuscript substantially, extended the underlying data resource/atlas set and thus believe it is now of much higher quality and therefore suitable for publication in Nature Genetics. Specifically, we have:

- **Performed extensive benchmarking** to improve usability and demonstrate that ArchMap decisions are based on optimized workflows for reference mapping. This included a benchmark to identify an appropriate threshold for the number of overlapping genes between the reference and query datasets that allow for a good quality mapping and benchmarking the optimal number of epochs for query-to-reference mapping to remove batch effects while conserving biological variation.
- **Fixed usability issues** with ArchMap that were **raised by reviewers** and others that were identified after extensive **internal testing** with a group of naive testers from 3 different labs. This includes improved error reporting, a contact email to provide support, automatic error logging in the ArchMap database to allow for rapid troubleshooting, demo datasets for each atlas, and clear documentation including step-by-step tutorials and FAQs.
- **Addressed privacy concerns** by enabling uploaded atlases to be private. We have provided two ways of accessing ArchMap locally: (1) enabling the full backend and frontend to be runnable locally or (2) enabling local reference mapping and annotation through a full code Python notebook.

Best regards,
Fabian, Malte, Mo, on behalf of all authors

In the following, reviewers' comments appear in *blue*, our point-by-point answers appear in **black**, figures we have added in the manuscript or supplementary are placed below the

relevant response and passages altered in the manuscript are written inside quotation marks and indented in the response letter.

Editor

Response to Reviewer 1

In this manuscript, the authors describe ArchMap, a web tool for mapping single-cell query data to a collection of reference atlases using one of several previously published mapping models and classification methods. The user uploads their data to a private location, but can choose to share the results with other users. The aim is that the tool should be usable by scientists without requiring them to write complex code.

Overall I found the manuscript well written, the website appealing and the documentation of the tool quite extensive. Below are my specific comments based on reading the manuscript and trying the tool with an example data set.

We would like to thank the reviewer for their kind words and their effort for improving this manuscript and tool.

1. I found the introductory paragraphs (lines 31-81) a bit hard to follow and somewhat lacking linear structure. The same statements are made several times (e.g., that coding limits usability for non-computational users), and properties of ArchMap are described (line 46) before it is properly introduced (line 55). I think this section could be made easier to read by making some rearrangements of the text.

We have reviewed the introductory paragraphs and have reorganized the text for clarity to make sure that ArchMap is introduced before describing any properties. We have also removed repetitive text, specifically pertaining to the statements on coding limiting usability. The first part of the introduction now reads:

> "Large-scale integrated single-cell reference atlases have become key for understanding inter-cellular variation and achieving consensus on cell type nomenclature[1–4]. A central use of these reference models has always been to gain insights into new data by mapping it on top of the atlas using transfer learning[5–9], similar to using genome references in genomics[10]. However, leveraging these methods requires knowledge of programming and nowadays often GPU compute and, even with this know-how, setting up the complex pipelines required for atlas use is time-consuming and potentially prohibitive for users.

To address this limitation, tools that simplify the use of these methods have been developed; however they are either limited in their focus or analysis capabilities or restricted by paywalls. For example, Azimuth[7,8] provides a graphical user interface (GUI) allowing atlas access to its users to project new single-cell data; however, the platform lacks collaborative features and mapping can only be done using methods from Seuratv4. This prohibits free, no-code comparison of reference mapping methods and flexibility to choose the optimal reference mapping approach. Fastgenomics[11] provided reference mapping to the HLCA; however, access is no longer freely available. scGPT Hub[12] and CellTypist[13] are GUIs that can also be used for reference mapping and cell type annotation, respectively; however, scGPT is a foundation model pre-trained on diverse datasets and only fine-tuned on the user's desired reference and query dataset and thus may introduce certain biases and remaining batch effects from other non-relevant datasets. In fact, foundation models have been shown to perform worse for integration tasks compared to scVI[14] and are also more computationally expensive, specifically when fine-tuning. Furthermore, celltypist on its own does not allow for removal of batch effects and thus another method or tool would need to be used to do the integration of the query into the reference.

ArchMap (Architecture Mapping; https://www.ArchMap.bio) is a free, no-code query-to-reference mapping framework that provides compatibility for python-based mapping methods and includes out-of-the-box cell type label transfer, uncertainty estimation, and collaborative analysis features. The fully automated approach makes query mapping accessible to users with and without coding and machine learning expertise through its intuitive GUI and access to a full code tutorial for more advanced users."

2. In the introduction, it is noted that for Azimuth, 'mapping can only be done using methods from Seuratv4. This prohibits compatibility to atlases integrated using strictly python methods'. Later, ArchMap is described as providing a framework that 'extends to python-based mapping methods'. To me, this indicates that also the previous R/Seurat-based methods should be supported (hence the 'extends'). However, as far as I can understand this is not the case, and ArchMap rather provides an alternative, for specific python-based methods. I think this should be made clearer.

We agree with this comment and have made the appropriate changes. That is we have changed the text to the following:

"ArchMap (Architecture Mapping; https://www.archmap.bio) is a free, no-code query-to-reference mapping framework that provides compatibility for python-based mapping methods and includes out-of-the-box cell type label transfer."

I tested the tool with the 'pbmc3k' dataset provided in the 'TENxPBMCData' R package. More precisely, I obtained and converted this dataset to h5ad format as follows (R v4.4.1, Bioconductor 3.19):

```
> x <- TENxPBMCData::TENxPBMCData("pbmc3k")
> x$batch <- x$Individual
> colData(x) <- colData(x)[, c("Sample", "Barcode", "batch")]
> zellkonverter::writeH5AD(x, file = "pbmc3k.h5ad", X_name = "counts")
```

After this, opening the object in python I can confirm that x.var_names are indeed human Ensembl IDs.
>>> x.var_names
Index(['ENSG00000243485', 'ENSG00000237613', 'ENSG00000186092',
...
I then attempted to upload this object to test it with the human PBMC atlas, the scPoli model and the KNN classifier.

3. For the atlas and the model selection, hovering over an option shows me the 'select' option, but even after clicking on it, it does not change to 'deselect' (it still says 'select'), as opposed to the classifier selection (which now says 'deselect'). I was initially unsure whether I had in fact selected the atlas/model.

We appreciate the reviewer's efforts to test the platform. This feedback has been very valuable for us to improve the tool. The issue of clarity upon model selection has now been fixed so that on selection both atlas and model cards buttons change to "deselect" as with the classifier card.

4. After uploading the data set, the processing fails with an error "zero-dimensional arrays can not be concatenated". It is not clear to me where this comes from, and if it indicates an issue with the uploaded data. Due to this error, I was not able to explore the tool further.

Thank you very much for raising this issue! This issue was coming from the stored h5ad file of the PBMC reference. That is, the array needed for concatenation in the reference was saved as a sparse array. This issue has now been fixed in the reference dataset and we have successfully tested mapping the given dataset (which we have obtained using the same steps that the reviewer has provided above) and have tested that this issue does not occur with other reference datasets. In addition, we have automatically logged all errors in the ArchMap database for all user mappings to be able to fix any issues encountered by users when mapping their data in ArchMap. This allows us to fix any errors without the user needing to inform us through GitHub issues or through the website contact form. Using this method, we have received no new errors for all user mappings (a total of 28) from 18.10.2025 to 19.11.2025 (the time of writing this).

5. It would be very helpful to have guidance on how to properly prepare the input data starting from a SingleCellExperiment or Seurat object - e.g. in terms of which checks should be performed before saving the object as an h5ad file. Having a way to validate the h5ad file locally before uploading would also be helpful (alternatively, that the object would be validated before the processing starts and that any deviations from the expectations would be clearly stated).

This is a very helpful suggestion! We have now provided a tutorial that checks the compatibility of a SingleCellExperiment or Seurat object with the ArchMap query requirements. Additionally we have provided a sheet that references all query datasets that have been successfully tested by the ArchMap authors. This includes datasets that were converted from RDS format to h5ad using this tutorial.

Additionally, we worked hard to improve the clarity of error reporting relating to dataset formatting issues and to improve our ability to provide support in these situations. Based on the errors reported by the reviewers, and errors that have come up in additional internal testing of cross-language conversion of query datasets, we have implemented dedicated error reporting and mitigation strategies. Specifically, we triage errors into two groups: issues with the query data that cannot automatically be changed in the pipeline and general issues where an h5ad file cannot be read by scanpy. In the first case, a detailed error message is sent to the user on how to fix their query dataset before mapping. In the second case, an error message is shown to the user requesting that a screenshot of the error is sent to archmap.bio@gmail.com or through the contact form on the website. Also, as mentioned above, if a user encounters an error, we automatically log this error in our database. This has allowed us to fix any unforeseen issues without the need for users to contact us directly. This has allowed us to reduce the error rate of mappings to zero in the last month, even with mappings being run on almost a daily basis by users in this time period. Additionally, we have provided FAQs on the website in case the user is unsure how to fix their query data format. Users can also make use of our tutorials to check the compatibility of user data where the data either has an RDS format or an h5ad format.

6. After failing to upload my own data set, I wanted to see if there was a demo data set provided with the application. However, this does not seem to be the case currently (there is a 'Select Demo Dataset' header, but it says that there are no existing datasets available). I think this would be helpful to provide as well, for users to be able to explore the platform.

We apologize for this oversight and have now provided 38 demo datasets, covering all atlases, including scvi hub atlases. Citations for all demo datasets can be found in the ArchMap docs

7. Currently the tool requires the user to upload their data to the Google Cloud. Despite security assurances, I imagine it may still deter some users or organizations. I was just wondering if, with the advances of WebAssembly tools, the authors envision a way that the computations could be performed in the user's browser at some point.

Thank you very much for this feedback! We are always eager to make ArchMap relevant for a larger user base. In theory, WebAssembly could be leveraged to use the capabilities of ArchMap without sending user data to an external server. From our research so far into how this could be implemented, we have come across instances where WebAssembly has been used for model inference in the browser, but not for model training. We expect that such an implementation will come with major challenges such as working around memory constraints (max 4Gb in the browser) and performance overhead from the lack of direct access to system resources. Particularly, the memory constraints will put even further limitations on query size and mappings and downstream calculations (such as calculation of UMAP embeddings) for references with a large number of features would in general not be possible.

With this in mind, we have provided a work around for users who do not trust the upload of their data to Google Cloud. In particular, we have added detailed information on how a user can install and run ArchMap locally on our GitHub. We understand that this is quite a

technical task and may deter users that are not familiar with Node.js. Thus, we have also provided a tutorial as a python notebook. This tutorial provides the user with the exact mapping pipeline that is used in ArchMap, but can be run locally. The user only needs to create an environment by running a provided bash script and then running the notebook. By default, the tutorial is set to map a provided query to the HLCA. If the user would like to use their own data and a different reference, they can download the desired reference from ArchMap and change the input and output paths in the tutorial notebook. We have added the following to the main text to reference this:

> “Expert users can also run the ArchMap pipeline locally by hosting the web application themselves. Expected costs amount to 200 Euros a month for multiple mappings a day for Google Cloud Services (including storage and compute). A full code Python notebook has been provided for running the pipeline locally as an alternative (Methods)."

8. The paper mentions 'access to full code tutorials for more advanced users'. Could you expand on this statement? Does this refer to tutorials describing how to perform the whole process locally (this would be cool)? I was not able to find a reference to such tutorials. The notebooks that are referenced cover things like addition of the batch variable and separating/concatenating large data sets, which would be required from all users to make their dataset compatible with the ArchMap assumptions.

Thank you for your feedback! We had created a tutorial, but did not reference it in the docs. We apologize for this oversight! We have now provided a step-by-step guide in the documentation on how local mapping can be done using this tutorial. The linked tutorial provides steps on mapping your own data to the HLCA, with demo query data provided. This tutorial can also be used to map to any other reference using a user’s query data with minimal adjustments to the tutorial notebook (e.g. changing of input/output file names).

9. Generally, 'ensemble IDs' should be 'Ensembl IDs' (in the manuscript and on the webpage)

This has been changed in both the manuscript and webpage

10. Line 396: upun -> upon

Thank you. This has been changed in the manuscript

Response to Reviewer 2

The authors present ArchMap a web-platform for atlas integration and cell-type label transfer. This is an important contribution to democratize access to single-cell reference models by providing advanced reference mapping functions to a wider user base.

Thank you for the kind words and the effort put into making Archmap better. We appreciate this effort and have done our best to address the feedback provided.

General comments:
1. Currently, I wouldn't recommend using ArchMap due to technical issues. I am convinced that the authors can overcome these technical issues.
To highlight these issues. Despite several attempts, I couldn't overcome warnings about CSC sparse format and process datasets downloaded from CELLXGENE. The error message is not helpful as it doesn't highlight whether query or reference data is the issue here. For a web platform, I expect the developers to perform some conversions to reduce preprocessing effort. I was finally successful mapping a part of the HLCA data (see provided reviewer login for error messages).

Thank you very much for your valuable feedback! As the reviewer suggests, we have added further preprocessing measures and format checks to the ArchMap pipeline that include automatic formatting fixes of uploaded data from the user to conform to the ArchMap requirements. There are however cases where fixes cannot be added on the platform side and the user needs to make formatting changes themselves (for example, in the case the batch covariate key has not been labeled as "batch" in the obs key of the h5ad file; note we cannot guess what the batch covariate in a dataset should be). For this case, we have provided tutorials (for RDS or h5ad objects) to check the compatibility of user data with instructions on what to do if errors occur.
Regarding the error you were obtaining about CSC sparse format, we could unfortunately not find this error using the provided reviewer login. However, we were able to reproduce what we believe to be the same error that you were encountering. That is, that the concatenation of CSR and CSC matrices are not supported. This is particularly referring to concatenating sparse arrays on disk for large references. We have fixed this error by making sure that all arrays of our references are in CSR sparse format and by automatically converting all arrays of user queries to CSR format upon upload.

To catch all errors of which we are still unaware, we have added a prompt to the user to submit a screenshot to our support email address (archmap.bio@gmail.com) that is triggered when an error arises that we do not have a specific error message for (see response to reviewer comment #1.5 above for more details on internal error handling in the pipeline). In addition, we have implemented automatic error logging in the ArchMap database for all user mappings to be able to fix any issues encountered by users when mapping their data in ArchMap. This allows us to fix any errors without the user needing to inform us through GitHub issues or through the website contact form. Using this method, we have received no new errors for all user mappings (a total of 28) from 18.10.2025 to 19.11.2025 (the time of writing this). We would like to note that these mappings in this time period are all from external users (not including researchers we have recruited for beta testing), showing the increased robustness and usability of the website.

2. I uploaded a new trained scVI model together with data without any count data (minified format from scvi-hub). The atlas was uploaded but I couldn't see any report (scIB metrics or similar) and after logging out the atlas seems publicly available (marked as under revision). Allowing the atlas uploader a first look at the quality metrics would circumvent this. The quality metrics (scIB metrics etc.) should be accessible to the uploader.

We appreciate the comment that the atlas uploader should get the opportunity to first check the ArchMap atlas quality assessment. To provide some context: to evaluate an atlas, scIB metrics are run for the atlas and 2 other comparison methods to provide context for the obtained metrics. This can take a bit of time to run and thus it cannot be provided directly at atlas upload time. However, we think we have found a good solution to address the issue regarding the missing benchmarking report. Upon upload of an atlas, the user can now automatically run a benchmarking analysis (details on how this is done on the website is given in the documentation) to assess the quality of their atlas integration and compare it to other integration methods. After benchmarking the user can download the report from the website.

To communicate this in the paper we have added the following text to the methods section:

> "A user-uploaded atlas must first go through a revision process before it is publicly available on the website. The revision process consists of the following evaluation steps: (1) benchmarking the user uploaded model used for integration with scVI, scPoli with prototype learning, scPoli without prototype learning, and PCA (no batch effect removal). Benchmarking is done using scIB metrics[4] (2) Comparing the KNN classifier F1 scores between classifiers trained on the latent representations taken from the user integrated model, scVI, scPoli with prototype learning, scPoli without prototype learning and PCA. (see documentation for full details of the benchmarking process). Once the atlas has been successfully benchmarked, the user will be able download the benchmarking report from the website and can send this report to the ArchMap team at archmap.bio@gmail.com. This report contains only the benchmarking evaluation as described in points (1) and (2) above. Thus, no private data is shared through email. Once the report has been successfully reviewed to ensure integration quality, the atlas uploader can choose whether they want to make their atlas available to the public for mapping or to keep their atlas private.
> "

3. For all scvi-hub models, no classifier can be selected and even scANVI doesn't support classification. I wasn't able to test these models as training led to the error 'Native'.

Thank you very much for this feedback! Classifiers for all scvi-hub models can now be selected (Fig R1) and the error 'Native' has been addressed and fixed.

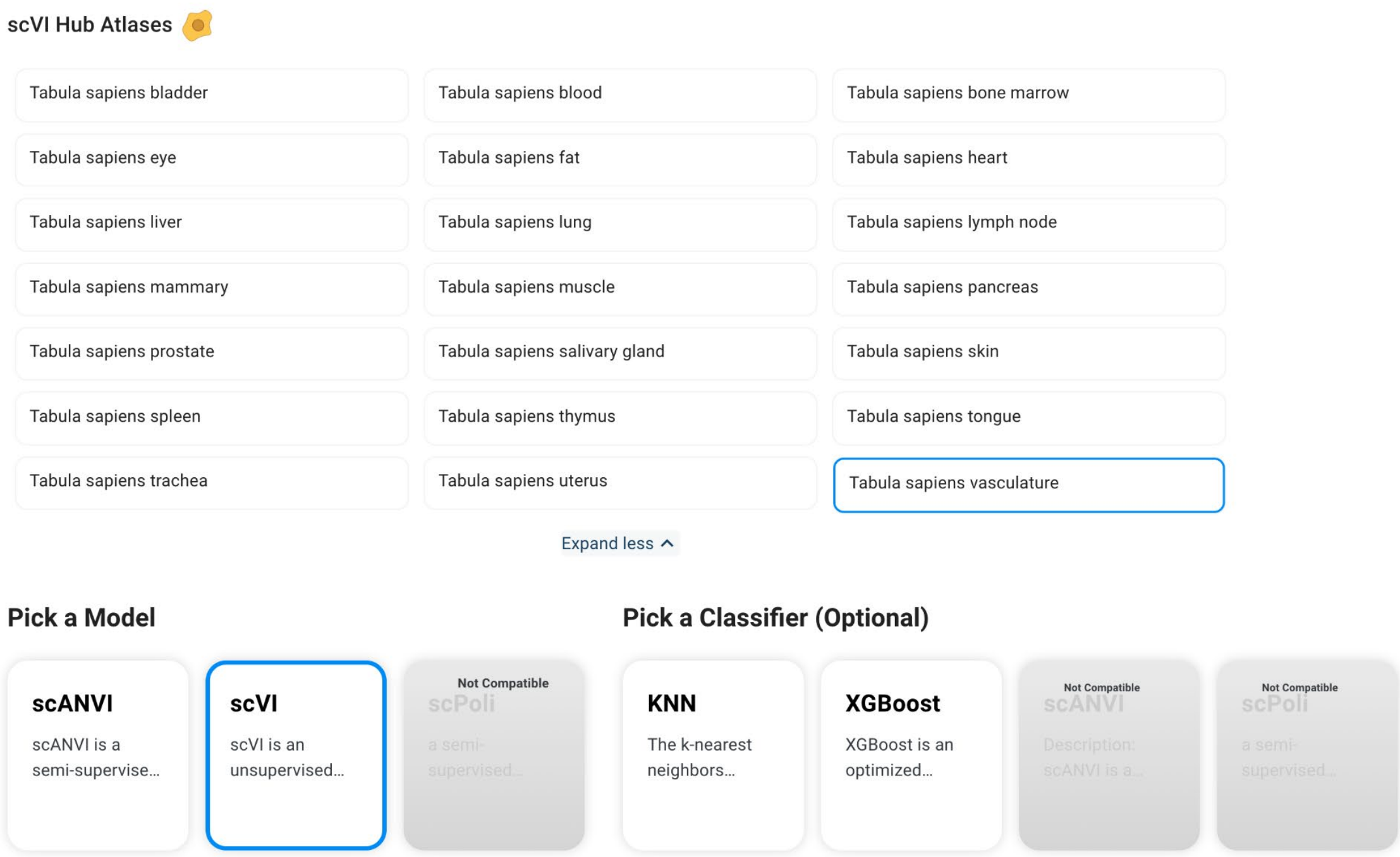

Figure R1: A screenshot of scvi-hub atlases with model and classifier selection that is now possible on ArchMap.

In addition, I have the following concerns.

Privacy concerns

4. The authors highlight data privacy for uploaded query datasets and provide a solid solution. However, it seems that all reference atlases are hosted publicly. Is there an option to have private reference atlases?

We appreciate this concern and have now added an option to set your uploaded atlases to private/public. By default, when an atlas is uploaded, the atlas is private and can only be accessed by the atlas uploader. Once uploaded the user can switch the atlas to public or

private any time as shown below (Fig R2).

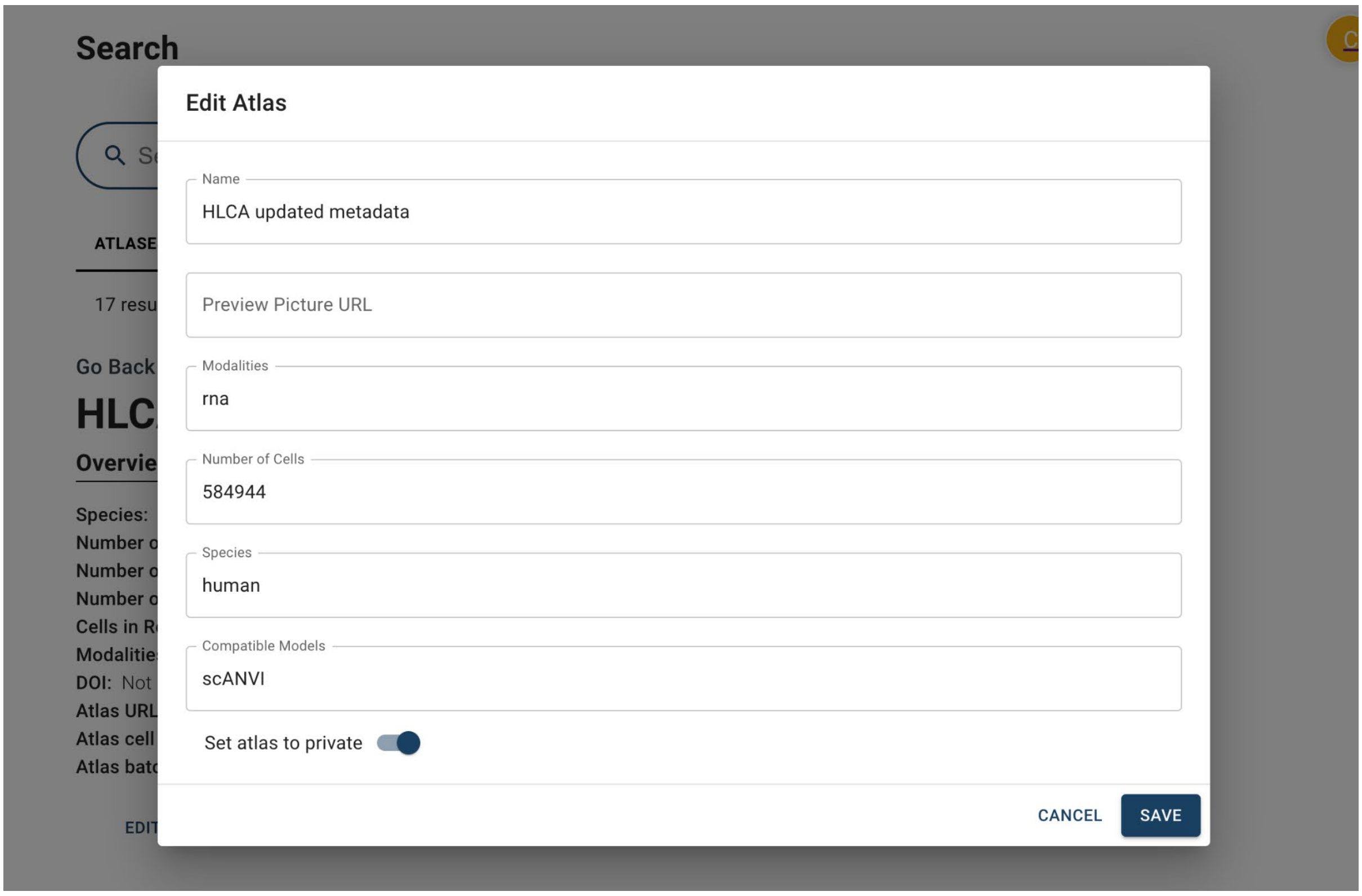

Figure R2: A screenshot of the Edit Atlas feature on ArchMap where the user can set their uploaded atlas to public or private.

We have additionally included the following text in the manuscript to communicate this:

> "If all quality checks are passed, the atlas uploader can choose whether they would like to make their atlas publicly available on the ArchMap platform or keep the atlas for private use (Fig. 1a; Atlas upload revision in Methods)."

As we detail in our response below (5.), we have also provided a tutorial in the form of a Python notebook that allows the user to map their query dataset to any reference atlas (including atlases not yet on ArchMap). This allows the ArchMap pipeline to still be leveraged in special cases where users are not able to upload data to google cloud storage, such as in a commercial setting.

5. Is the only option to self-host the full ArchMap webpage?

As mentioned above, self-hosting is not the only option to preserve atlas privacy. However, we have also added this option to support users that want to set up fully private ArchMap usage. Specifically, we have added a tutorial that allows the user to access the mapping process from a jupyter notebook. A conda environment is provided which can be automatically activated using a bash script. The details are given in the documentation.This mapping process is exactly the same as the mapping process on the ArchMap website.

6. The developers provide guidance on setting up a custom ArchMap web interface on their GitHub README. Could they argue about monthly costs and time to set up an interface?

The time taken to set up the interface may depend on familiarity with tools and technologies such as Git, Docker, MongoDB, and Node.js and system administration skills. If there is no previous knowledge, it may take a week to set up the interface and get familiar with the tools and services used. Furthermore, if the user would like to use Google Cloud Services including storage and compute services, to manage the web application, we predict, based on experience, that there may be a monthly cost on average of 200 Euros if the interface is used regularly, specifically approximately one to two mappings per day (based on the current usage stats of ArchMap).

We have also made available a tutorial, as mentioned previously, in the form of a Python notebook which encompasses the full mapping pipeline of ArchMap. We believe this is the best alternative for users that do not want to host the website fully locally as the tutorial only requires the user to clone the ML repo, run a bash script to activate the environment and change input and output path names in the beginning of the notebook to their desired atlas, query and results files.

We have commented about monthly costs in the manuscript and have referred to the ArchMap full code python tutorial notebook as an alternative to running the pipeline locally for users that do not need to host the full ArchMap website locally or may not have the technical expertise to do so:

> "Expert users can also run the ArchMap pipeline locally by hosting the web application themselves. Expected costs amount to 200 Euros a month for multiple mappings a day for Google Cloud Services (including storage and compute). A full code Python notebook has been provided for running the pipeline locally as an alternative (Methods)."

Model training

7. The author benchmark splitting datasets into separate batches highlighting preservation of cell-type annotation. This is a very coarse measurement especially as small deviations from identity are not well-displayed using a confusion matrix. The authors should compare reconstruction loss or neighborhood preservation in latent space between both scenarios.

We appreciate the reviewer's request for further benchmarking to ensure our proposed approach aligns with best practices. We have now additionally compared the two scenarios using scIB metrics that are widely used in the field to evaluate data integration performance. These metrics allow for further analysis into the mapping quality of both scenarios, including assessment of biological conservation and batch correction. As shown in the newly added Extended Data Fig. 3c below, we see that there is very minimal change between mapping individual batches (separate) compared to mapping the full query (combined). Indeed, we only see a difference of 0.01 in both PCR comparison and KMeans ARI, however this is not the case for other metrics and could therefore be a result of stochastic effects. Based on these results, we do not expect the user to encounter a decrease in mapping quality from mapping batches separately.

a. **b.**

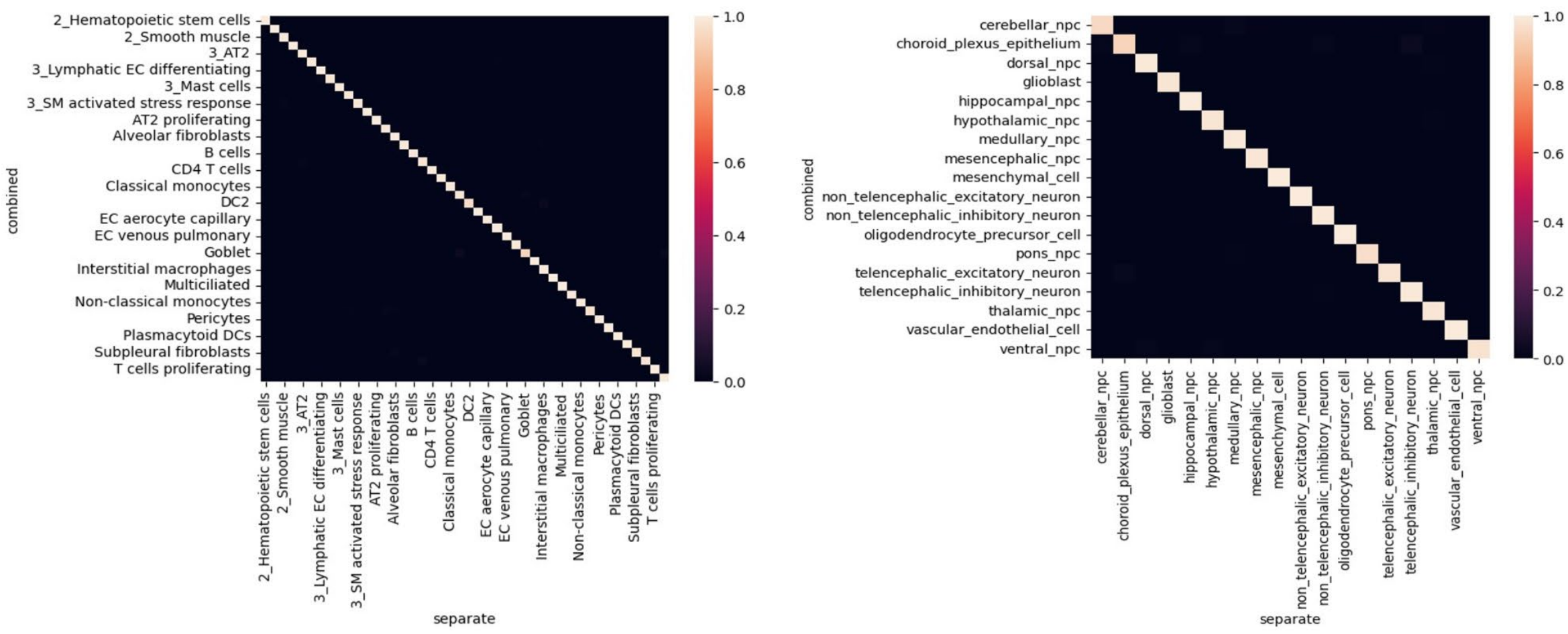

c.

Method	Bio conservation: KMeans NMI	Bio conservation: KMeans ARI	Bio conservation: Silhouette label	Batch correction: KBET	Batch correction: Graph connectivity	Batch correction: PCR comparison	Aggregate score: Batch correction	Aggregate score: Bio conservation	Aggregate score: Total
**Combined**	0.70	0.45	0.54	0.41	0.92	0.20	0.51	0.56	0.54
**Separate**	0.70	0.44	0.54	0.41	0.92	0.19	0.51	0.56	0.54

Extended Data Fig. 3 Comparing mapping and label transfer performance of separately mapped query batches vs. a single query mapping containing all batches

Comparing label transfer performance of separately mapped query batches vs. a single query mapping containing all batches for the **a,** HLCA and **b,** HNOCA. Both methods give the same results for cell type label transfer. **c**, Comparing scIB metrics between the two scenarios mapped to the HLCA.

8. The authors provide interpretation for various quality scores. However, I am not aware that these scores and especially cut-offs are well established in the literature. The authors should benchmark this aspect. E.g. the authors argue that if 20% of the genes are missing (depending on the location in the documentation 15%) the results should be further scrutinized. Instead of giving a fixed value, another approach could be to subset the reference data to the overlapping genes and benchmarking performance on a corrupted small subset of the reference dataset. This won't add much computational cost but would give better guarantees.

Thank you very much for noticing this discrepancy, the feedback, and the suggestion. This comment really forced us to re-evaluate our assumptions from preliminary tests and we were quite surprised by the results from the systematic benchmark we did.

Firstly, we have identified the inconsistency, performed an extensive benchmark, and made sure a consistent recommendation is given in the text and the documentation. Secondly, as deep learning algorithms perform better with more training data (and we have observed this

several times with scArches) and the batch effects of reference datasets have already been seen by the model, we feel that using a small subset of the reference data to investigate mapping performance under missing genes for each query dataset is unlikely to be generalizable to other query datasets and therefore not helpful for users.

To address this very relevant comment and still be able to provide a guideline for the number of missing features, we have extensively tested the effects of missing genes using different thresholds and gene selection approaches using the HLCA with a new query dataset. This benchmark is explained in the new Supplementary Note 1. In brief, we harmonized cell type labels for a new dataset, and used 3 different methods to select genes to filter out to test reference mapping. These 3 methods represent an omission of random genes, a best case scenario (least informative genes are dropped), and a worst case scenario (most informative genes are dropped). Reference mapping was then evaluated by testing label transfer performance (F-1 score). From our results we see that both methods of taking the most informative genes and a random sample show that 50% overlap already allows for clustering of cells to distinct areas on the reference embedding (Supplementary Figure 1). Subsetting to only the most informative genes, however, is not a realistic scenario as mapping with a 50% overlap between query and reference does not guarantee that all genes are informative of the cell types in the query. Furthermore, compared with taking a random sample, considering only genes that are least informative of cell type provides a lower bound to mapping performance. Thus, this method was used to develop a guideline for percentage overlap based on cell type classification F-1 scores (Supplementary Figure 2). In particular, based on our benchmark, we have updated our guideline to recommend that the overlap of genes between the query and reference should be no lower than 80%. At this level we expect that for 80% of the cell types, the F-1 score will fall within 15% of the optimal performance (Supplementary Figure 2).

We refer to the benchmarking experiment and Supplementary Note 1 in the methods section of the manuscript, which has been copied below:

> "During mapping, only genes used to build the reference atlas model can be used to map a query dataset to this atlas, requiring the genes of query datasets to be subsetted to match the atlas gene set. To better understand the effect of this subsetting on query to reference mapping, we benchmarked query mapping quality under varying overlap of profiled genes in the HLCA reference (see Supplementary Note 1). This experiment has allowed us to provide a more informative guideline to users mapping their query to a reference atlas on ArchMap. Based on our benchmark, we recommend that the overlap of genes between the query and reference should be no lower than 80%."

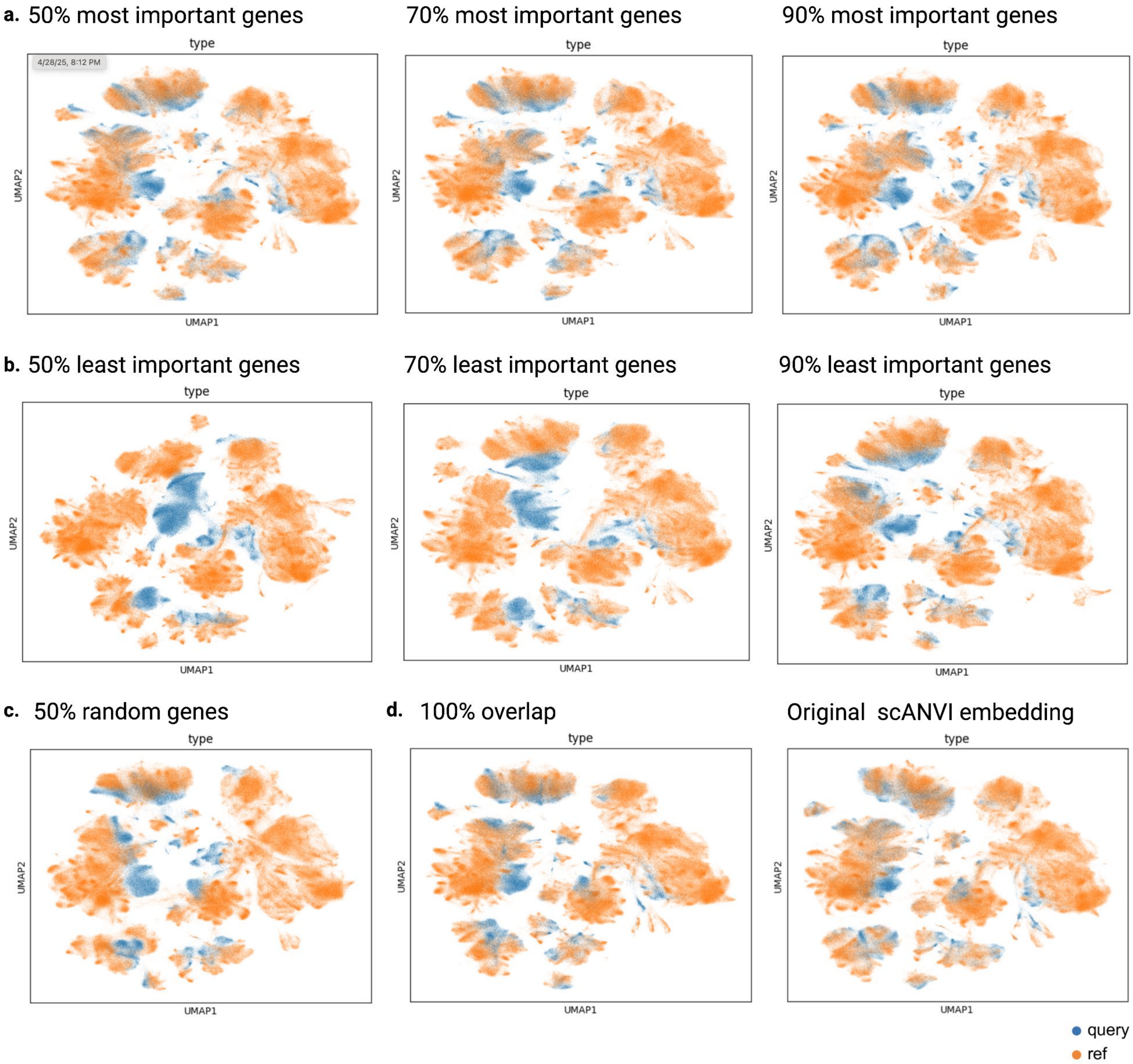

Supplementary Figure 1: Benchmarking the number of overlapping genes between reference and query

Benchmark of the number of overlapping genes between the HLCA reference and a healthy query with harmonized cell type labels taken from the HLCA extended atlas. The subset of overlapping genes was chosen using 3 different methods: **a.** removing least informative genes (best case scenario), **b.** removing most important genes (worst case scenario), and **c.** an omission of random genes. Gene importance scores were obtained from an XGBoost classifier trained on the query count data for cell type prediction. **d.** UMAP showing the mapping results using 100% overlap (left) and UMAP showing the embedding from the HLCA extended atlas subsetted to the HLCA core and query data used in this benchmark (right) showing the variability under rerunning the mapping.

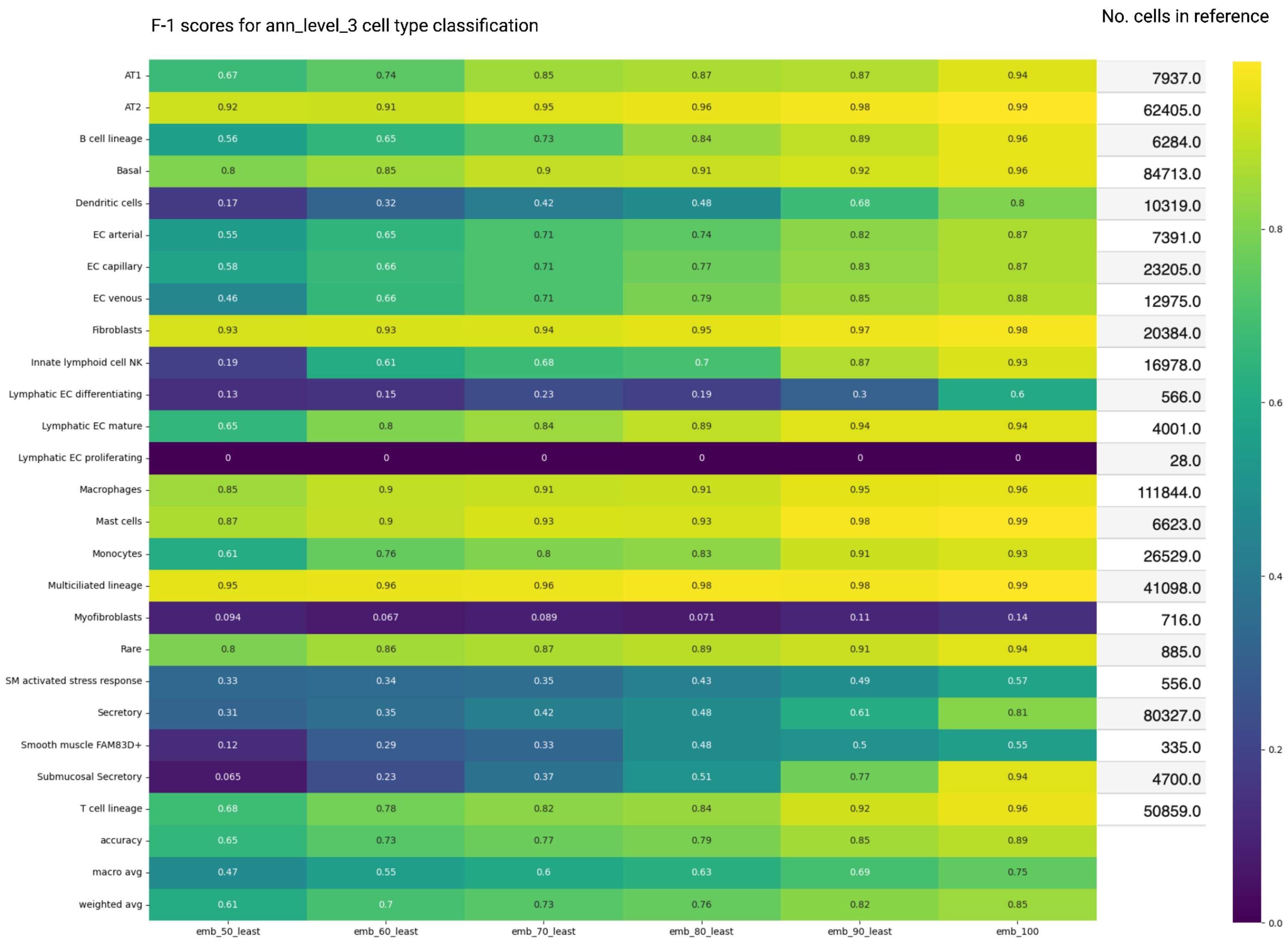

Supplementary Figure 2: Evaluating cell type classification F-1 scores for worst case scenario (keeping least important genes for mapping)

Benchmark of the number of overlapping genes between the HLCA reference and a healthy query with harmonized cell type labels taken from the HLCA extended atlas. F-1 scores evaluate cell type classification performance using a KNN classifier trained on the reference embedding. Genes were chosen based on the worst case scenario (least informative genes for classifying cell type) to obtain a lower bound on a threshold of the number of overlapping genes needed for mapping.

9. The authors argue about multi-modal data (including ATAC) and have an API for totalVI models in GitHub. Is totalVI supported in ArchMap?

We initially focus on scRNA-seq only - mostly because nearly all big integrated atlases are unimodal. But, indeed, atlases currently being developed, specifically as part of the Human

Cell Atlas, such as the human lung cell atlas version 2 or the immune atlas, are incorporating other modalities such as CITE-seq and spatial transcriptomics data. So while TotalVI is currently not supported in ArchMap, it is a part of our outlook to incorporate multi-modal integration in the pipeline. We have included the following in the main text of the manuscript to communicate this clearly:

> "With the inclusion of other modalities such as CITE-seq and spatial transcriptomics data in the new generation of atlases currently being developed, we foresee ArchMap growing to accommodate multi-modal reference mapping by extending to incorporate deep learning frameworks such as TotalVI[28] and MultiMIL[29]"

10. The limitation to 50k cells will likely be stricter on ATAC data due to dataset size. How many cells can likely be mapped for multi-ome data?

Although we have been able to increase the cell limit to 200k, we agree that with the addition of ATAC data, the limit will likely be cut by more than half, due to ATAC data having a much larger number of features used for mapping in MultiMIL for example. The current limit is set due to the limitation on memory and disk space (32 Gb) in google cloud run.

While the addition of ATAC query data is part of our outlook, and we do not yet support this, we are happy to brainstorm this situation here: A potential solution would be to move to google cloud AI services in the future to allow for an increase in memory allowance. This is costly, but would be warranted by a high demand in ArchMap as a go-to tool for query to reference mapping. By taking this step, we would be able to mitigate stricter cell limitations and even increase this limit. However, we foresee implementing this extension only once the number of multiome or ATAC atlases grows, which would increase the relevance of mapping to these atlases.

11. I found that even for 500k cells scArches was initialized but never finished. An error should be raised for large datasets.

As the original limit was stated as 50k cells and not 500k on the website, we assume that the reviewer has meant mapping with 50k cells. We apologize for this oversight and we have tested all atlases to make sure that mapping works with the limit that we have now stated in the manuscript, the website and the docs, which is 200 000 cells. We have been able to increase the cell limit to 200,000 cells and, as the reviewer suggested, we have incorporated a message in the pipeline that is sent to the user when they exceed this limit upon upload. We have been able to increase the limit by changing the Google cloud compute service to google cloud run jobs, which allows users to run a computation indefinitely (this was not the case for google cloud run, used previously, which had a time limit of one hour). Google cloud run jobs unfortunately still have limitations on memory and thus we still have to set a limitation on the number of cells that a user can upload to allow for a successful run.

12. The authors state that depending on the atlas ensemble gene IDs or gene symbols are expected. The conversion between those should optimally happen on-the-go within ArchMap to reduce computational burden.

While the conversion between ensembl gene IDs and gene names depends on the genome version used by a reference and query dataset (and is therefore dataset dependent), we do feel that the conversion for the highly variable genes typically used for mapping should be quite stable, which merits an internal conversion proposed by the reviewer. Thus, we have incorporated an automatic conversion for the query dataset depending on whether the atlas has ensemble gene IDs or gene symbols. We have obtained Biomart mapping tables for the conversions of both mouse and human following this tutorial. In order to give the user the opportunity to improve on this conversion in case of insufficient performance, we let the user know that this conversion was done internally, as shown in the screenshot below (the warning is in green). We have added this explanation to the methods section.

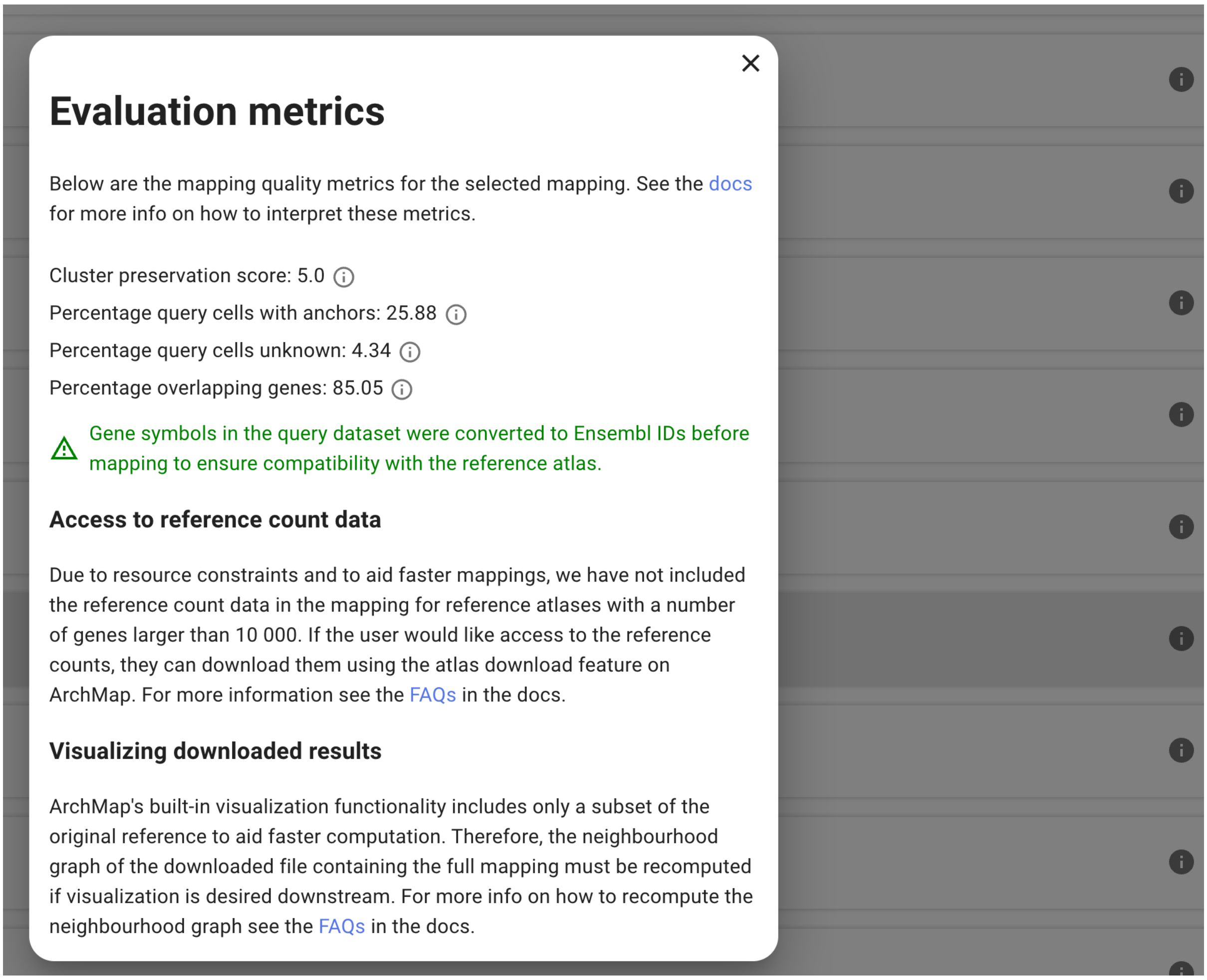

13. The authors provide KNN and XGBoost as classifiers and argue that if the uncertainty is high XGBoost should be used. How do they reach this statement?

There seems to be a misunderstanding underlying this comment, as we did not intend to make such a statement. We believe that the following sentence in our documentation may have led to this misunderstanding:
If a large percentage of your query cells are labelled "Unknown", we recommend to compare label transfer results with the XGBoost classifier.

The reasoning behind this sentence is to communicate that if the classifier that has been used gives label transfer results with high uncertainty, then other available classifiers for the atlas should also be used to compare results.

We have reworded the sentence to the following for better clarity in the documentation:
If a large percentage of your query cells are labelled "Unknown" with your chosen classifier, we recommend to compare the label transfer with other available classifiers to determine if uncertainty can be improved.

14. scANVI is benchmarked on the published HLCA model. However, their developers raised an issue with the classifier and fixed the model more recently: https://docs.scvi-tools.org/en/stable/tutorials/notebooks/scrna/scanvi_fix.html. [docs.scvi-tools.org] Benchmarking should be performed after retraining on the same dataset for scANVI and scPoli in extended figure 2.

Thanks a lot for pointing out this oversight! For HCA-approved reference models we strive to use the extensively evaluated and approved versions of the atlas rather than recreating a model ourselves. Since retraining of the HLCA atlas will destroy a lot of extensive performance evaluation and community based annotation, we have instead removed the native scANVI classifier from ArchMap for HLCA. We have incorporated our reasoning behind not reintegrating any atlas uploaded to ArchMap in the main text of the manuscript as follows:

> "All reference atlases available in ArchMap are the extensively evaluated, published and, where available, consortium-approved versions (e.g., by the Human Cell Atlas[27]; HCA) of the atlases. We do not retrain models ourselves, as this voids the extensive performance evaluation and community based annotation that is typically done for HCA atlases."

15. For Euclidean and Mahalanobis distances as well as Presence Score the calculation doesn't become fully clear (value of 0.5 as cut-off doesn't make sense with the description). The authors should provide a formula or pseudocode.

Thank you very much for this feedback. We understand now that more detail needs to be given for how these scores are calculated. We have thus extended the methods section on these scores to improve clarity. We have also included the reason why 0.5 is chosen as a cut off during label transfer in the methods section. The text is copied here:

For the Euclidean and Mahalanobis uncertainty scores:

> "The Euclidean uncertainty calculated for a cell is based on the Euclidean distance between that cell and its 15 nearest neighbours. This score is used to help the user identify novel cell states in the query data. We have added a separate column, named "{cell_type_key}_prediction_{classifer_name}_filtered_by_uncert>0.5", in the output that labels cells as "unknown" above a certain uncertainty threshold. Here, cell_type_key is the key in the obs attribute of the cell type level mapped to and

classifer_name is the name of the chosen classifier (e.g. knn). The threshold is set to 0.5 and is based on the way the Euclidean uncertainty is calculated. The Euclidean uncertainty of the query cells is calculated using the weights of a weighted k-nearest neighbour classifier trained on the reference embedding, the same method used in both the original scArches[28] and HLCA[1] manuscripts. The KNN classifier is applied to the query embedding, where the distances to the 15 nearest reference cells are measured for each query cell. A Gaussian weighting function is used to assign more importance to nearer reference cells, and is given by

$$\tilde{d}_{ij} = \exp\left(-\frac{d_{ij}}{\sigma'_i}\right),$$

where d_{ij} is the Euclidean distance between the i-th query cell and the j-th nearest reference cell and $\sigma'_i = (2/\sigma_i)^2$ where σ_i is the standard deviation of the distances to the 15 nearest reference cells.

The weight w_{ij} corresponding to the j-th nearest reference cell of the i-th query cell is normalized by the sum over all K neighbors so that the weights for each query cell sum to 1:

$$w_{ij} = \frac{\tilde{d}_{ij}}{\sum_{k=1}^{K} \tilde{d}_{ik}}.$$

For each unique cell type label from the reference nearest neighbours, a probability for that label is calculated by summing the corresponding reference cell weights. The reference label with the highest probability is then used as the label for the query cell. To calculate the uncertainty for that query cell we take $1 - p$, where p is the probability of the reference label used for label transfer. Thus, we set a threshold of 0.5 since a probability below 0.5 is more uncertain than certain and no label stands out as a strong prediction.

For the case that users are interested in considering novel cells in the query dataset, we have also included in the output a column with all transferred labels for each query cell without any threshold used. We named this column "{cell_type_key}_prediction_{classifer_name}". This allows users to also obtain transferred labels for query cells with uncertainty greater than 0.5. Furthermore the "{cell_type_key}_uncertainty_euclidean" column can be used to view the exact uncertainty scores for all query cells.

The Mahalanobis uncertainty is based on the Mahalanobis distance between a query cell and the means of the Gaussian components from a Gaussian mixture model of the reference embedding, where the number of components is equal to the number of cell types. In particular, the Mahalanobis uncertainty is given by

$$\frac{1}{K}\sum_{k=1}^{K} \gamma_k(x) d_M(x, \mu_k),$$

where $d_M(x, \mu_k) = (x - \mu_k)^T \Sigma_\mu^{-1} (x - \mu_k)$ is the Mahalanobis distance between a query cell x and the mean of the k-th Gaussian component. Here, Σ_μ^{-1} is the inverse covariance matrix derived from the means of the K Gaussian components. Furthermore, $\gamma_k(x)$ is the probability of query cell x belonging to component k. This probability is the posterior probability calculated using Bayes' Theorem:

$$\gamma_k(x) = P(z = k \mid x) = \frac{\pi_k \mathcal{N}(x \mid \mu_k, \Sigma_k)}{\sum_{j=1}^{K} \pi_j \mathcal{N}(x \mid \mu_j, \Sigma_j)},$$

where $\mathcal{N}(x \mid \mu_k, \Sigma_k)$ is the probability density function of the k-th multivariate Gaussian distribution and π_k is the mixture weight of Gaussian component k.

Mahalanobis uncertainty accounts for potential correlation between the latent features of the cells. This is done by rescaling the latent features such that the covariance between any two correlated latent features is removed. If there are no correlations between the features, the distance d_M is equal to the Euclidean distance.

We compared the suitability of the Euclidean and Mahalanobis uncertainties in identifying disease-associated cell states in the use case presented in the main text of this manuscript (the mapping of an IPF dataset to the HLCA). From this analysis, we concluded that Mahalanobis uncertainty was the most appropriate to use as an uncertainty metric for this mapping (see Supplementary Note 2 for details). Based on these results, we recommend that the user make use of the Mahalanobis uncertainty to identify potential disease cell states. Although we found that the Mahalanobis uncertainty was more suitable for this use case, we have implemented both metrics in the pipeline as the Euclidean uncertainty has been validated in the HLCA manuscript and may be of interest as an uncertainty metric for users based on its more established use in single-cell reference mapping."

For the presence score:

"The presence score quantifies reference cell state representation in the query dataset, based on shared neighborhood structure between reference and query cells. Using a weighted k-nearest neighbor (WKNN) graph derived from the embedding space, we count how frequently each reference cell is identified among the k nearest neighbors (k = 15) of query cells. This results in an initial presence score p for each reference cell, defined as

$$p_i = \sum_{q \in Q} w(q, i)$$

where $w(q, i)$ is the WKNN weight of reference cell i in the neighborhood of query cell q.

To account for similarity structure within the reference dataset, the initial presence score for each reference cell is diffused using a random walk with restart performed over the reference cell transition probability matrix T, constructed from a reference KNN graph (k = 15):

$$p^{(t+1)} = (1-\alpha) T p^{(t)} + \alpha p^{(0)},$$

with restart probability $\alpha = 0.1$. The process is iterated for 100 rounds.

Presence scores are then log-transformed, clipped to the 1st and 99th percentiles, and normalized to $[0, 1]$."

16. In the anchor computation, the ratio of Anchor Cells is likely highly affected by the number of cells in query and reference data. Computation should be performed on a fixed number of reference and query cells.

We have carefully considered this comment and come to a slightly different conclusion. We agree that the ratio of Anchor Cells would be affected by the number of cells in query and reference data, and it makes sense to fix the number of query and reference cells used to calculate the score if the goal was to make the metric result comparable across mappings and references. However, we see that the main use of this metric is to convey the success of the mapping and the suitability of the chosen atlas as a reference for the specific query data. By fixing the number of query and reference cells considered for the score calculation, we may miss important information for assessing how well the query is integrated into the reference with all cells. For example, setting a fixed value for the number of reference cells would force a large number of reference cells to be removed for the calculation for a large reference. This may result in the metric underscoring the mapping quality, since some query cells may lose the anchors that they would have had if subsampling did not occur. On the other hand, setting a fixed value for the number of query cells would force a large number of query cells to be removed for the calculation for a large query. This may result in the metric overscoring the mapping quality, since more query cells that did not have an anchor before subsampling may be removed. Thus, to fix this we have stated the limitations of this metric on the website, in the paper, and in the docs here to make users aware that this metric is affected by the size of the query and reference and this should be considered if they want to do comparisons between mappings.
To communicate this in the paper we have added the following text to the methods section:

"We also note that the percentage of anchor cells is affected by the number of cells in the query and reference data. Thus this metric should not be used to compare mapping quality across query or reference datasets, but only across models and classifiers. The purpose of this metric is to convey the success of the mapping and the suitability of the chosen atlas as a reference for the specific query data."

17. The authors train models for 10 epochs. This is much lower than in tutorials for scArches. The number of epochs should be benchmarked (on datasets with less than 50k cells).

Thank you very much for this feedback! We have implemented a benchmark to evaluate the most fitting number of epochs for query-to-reference mapping. In the following we present our results (including the Extended Data Fig. 4), which have been added into the methods section of the paper. Since we have been able to increase the limit of query cells to 200 000, we have benchmarked using query data of approximately 70 000 and 118 000 cells from healthy and disease samples.

> “For all query-to-reference mappings, a fixed value of 100 epochs is used for fine-tuning the reference model. This number was chosen by running mappings of both healthy and disease query data, with approximately 70 000 and 118 000 cells respectively, to the HLCA and increasing the number of epochs from 1 to 1000. We evaluated the mappings using the reconstruction loss, scIB metrics and UMAP embeddings (Extended Data Fig. 4). We found that reconstruction loss for both query datasets plateaus after approximately 100 epochs (Extended Data Fig. 4a). Furthermore, for the healthy query, we see all scIB metrics continue to increase up until approximately 250 epochs, at which point the kBET and PCR comparison decrease slightly. After this decrease at approximately 500 epochs they then remain constant, along with all other metrics. For the disease query we see that past 100 epochs, we begin to see a decrease in PCR comparison, kBET, and KMeans ARI. Thus, for all mappings on ArchMap we have set the value for the number of epochs to 100 since for the majority of scIB metrics, we see no further improvement past this number (Extended Data Fig. 4b). Furthermore, for both the reconstruction loss and the UMAP embeddings, we see no change or improvement in integration when training for more than 100 epochs (Extended Data Fig. 4c).”

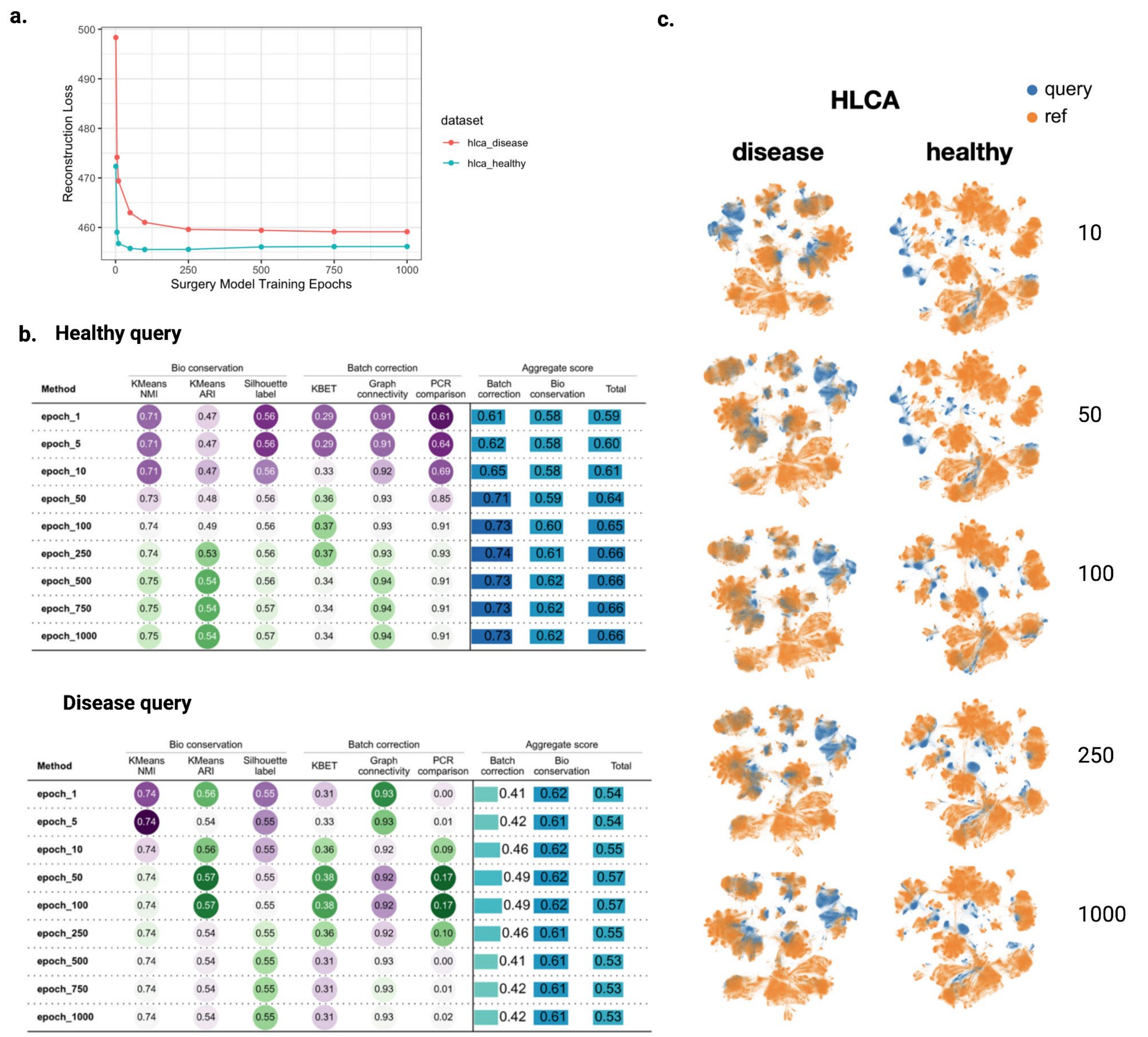

Healthy query

Method	Bio conservation: KMeans NMI	Bio conservation: KMeans ARI	Bio conservation: Silhouette label	Batch correction: KBET	Batch correction: Graph connectivity	Batch correction: PCR comparison	Aggregate score: Batch correction	Aggregate score: Bio conservation	Aggregate score: Total
epoch_1	0.71	0.47	0.56	0.29	0.91	0.61	0.61	0.58	0.59
epoch_5	0.71	0.47	0.56	0.29	0.91	0.64	0.62	0.58	0.60
epoch_10	0.71	0.47	0.56	0.33	0.92	0.69	0.65	0.58	0.61
epoch_50	0.73	0.48	0.56	0.36	0.93	0.85	0.71	0.59	0.64
epoch_100	0.74	0.49	0.56	0.37	0.93	0.91	0.73	0.60	0.65
epoch_250	0.74	0.53	0.56	0.37	0.93	0.93	0.74	0.61	0.66
epoch_500	0.75	0.54	0.56	0.34	0.94	0.91	0.73	0.62	0.66
epoch_750	0.75	0.54	0.57	0.34	0.94	0.91	0.73	0.62	0.66
epoch_1000	0.75	0.54	0.57	0.34	0.94	0.91	0.73	0.62	0.66

Disease query

Method	Bio conservation: KMeans NMI	Bio conservation: KMeans ARI	Bio conservation: Silhouette label	Batch correction: KBET	Batch correction: Graph connectivity	Batch correction: PCR comparison	Aggregate score: Batch correction	Aggregate score: Bio conservation	Aggregate score: Total
epoch_1	0.74	0.56	0.55	0.31	0.93	0.00	0.41	0.62	0.54
epoch_5	0.74	0.54	0.55	0.33	0.93	0.01	0.42	0.61	0.54
epoch_10	0.74	0.56	0.55	0.36	0.92	0.09	0.46	0.62	0.55
epoch_50	0.74	0.57	0.55	0.38	0.92	0.17	0.49	0.62	0.57
epoch_100	0.74	0.57	0.55	0.38	0.92	0.17	0.49	0.62	0.57
epoch_250	0.74	0.54	0.55	0.36	0.92	0.10	0.46	0.61	0.55
epoch_500	0.74	0.54	0.55	0.31	0.93	0.00	0.41	0.61	0.53
epoch_750	0.74	0.54	0.55	0.31	0.93	0.01	0.42	0.61	0.53
epoch_1000	0.74	0.54	0.55	0.31	0.93	0.02	0.42	0.61	0.53

Extended Data Fig. 4 Benchmarking number of epochs used for reference mapping
Benchmark of the number of epochs to be used for query-to-reference mapping using both disease and healthy queries mapped to the HLCA. Mapping quality is evaluated using the **a**, reconstruction loss **b**, scIB metrics using the scib-metrics package, and **c**, UMAP embeddings.

18. An additional field in the CELLXGENE session should be created in which all cells (query and reference have cell-type labels). Currently only query cells are labeled.

We have now included all query and reference cell type labels in one column for the label transfer (named "{cell_type_key}_prediction_{classifer_name})", where cell_type_key is the key in the obs attribute of the cell type level that was mapped to and classifer_name is the name of the chosen classifier (e.g. knn)).

Reproducibility using ArchMap:
19. After successful mapping, fine-tuned models should be accessible via download and not only the dataset.

Thank you for this useful suggestion. This feature has now been added. When a user downloads their results, the results are in a tar.gz file that includes both the dataset and the fine-tuned model. We have highlighted this in the main text of the manuscript:

> "Users are able to download an h5ad file containing the combined reference and query data with transferred annotations and the fine-tuned model used for mapping."

20. All reference atlases should be version-controlled. Scvi-hub models e.g. provide different versions that can't be accessed on ArchMap. Uploading reference atlases to scvi-hub could be a solution to allow reproducibility and easy access to reference models (huggingface provides version control). AWS or GitHub would be other options here.

Version control of available atlases is indeed a worthwhile target. The current archmap core atlases are linked to Human Cell Atlas approved reference atlases or larger atlas publications that we do not expect to change. Newer versions would currently be treated as separate atlases, as some people may still want to map to older versions that are typically inherently different embeddings. The scvi-hub atlas version compatibility is however a different matter. To solve the issue that different atlas versions on scvi-hub cannot be accessed on ArchMap, we have included a tutorial that walks the user through downloading different versions of an atlas from scvi-hub. The user can then upload this atlas to ArchMap to do their mapping on their desired atlas version.

To communicate this in the paper we have added the following text to the methods section:

> "On ArchMap, by default, users have access to the latest versions of all scANVI- and scVI-integrated scvi-hub atlases (upload of scPoli-integrated atlases is currently not available on scvi-hub). For the case where users would like to use an earlier atlas version on scvi-hub, we have included a tutorial in the ArchMap docs that walks the user through downloading different versions of an atlas from scvi-hub. The user can then upload this atlas to ArchMap to do their mapping on their desired atlas version."

Minor:

21. Scvi-hub models are supported in ArchMap. However, when navigating to https://www.archmap.bio/ [archmap.bio]#/references/atlases these don't appear but are only visible when creating a mapping: https://www.archmap.bio/ [archmap.bio]#/genemapper/create. This will create confusion for users that want to access a specific model stored in scvi-hub.

We have now added a specific tab for scvi-hub models in the references section on the website here

22. In the list of available tools for reference mapping the authors should refer to https://www.celltypist.org/ for Celltype annotation and scGPT hub for reference embedding using a foundational model. I think using specific reference models and query embedding provides advanced use cases beyond these two platforms.

Thank you very much for this suggestion! We have referenced both scGPT Hub and https://www.celltypist.org/ and have commented on their limitations as tools for query and

reference embedding and cell type annotation. That is we have included the following text into the manuscript:

> "scGPT Hub[12] and CellTypist[13] are GUIs that can also be used for reference mapping and cell type annotation, respectively; however, scGPT is a foundation model pre-trained on diverse datasets and only fine-tuned on the user's desired reference and query dataset and thus may introduce certain biases and remaining batch effects from other non-relevant datasets. In fact, foundation models have been shown to perform worse for integration tasks compared to scVI[14] and are also more computationally expensive, specifically when fine-tuning. Furthermore, celltypist on its own does not allow for removal of batch effects and thus another method or tool would need to be used to do the integration of the query into the reference."

23. Figure 1D IV doesn't have axis labels.

Thank you for pointing us to Figure 1D for further review. While we were initially confused as both UMAPs in the subfigure have axis labels, we noticed that the legend indeed is missing a label, which we assume the reviewer meant. This is now remedied.

24. The authors argue that scvi-hub provides access to unpublished models. Is it a requirement for ArchMap to have a preprint? What type of validation is performed on scvi-hub models? Are they less trustworthy in ArchMap?

The scvi-hub atlases that are publicly available that are compatible with scArches mapping include all compartments of the multiple-organ Tabula Sapiens atlas and the Human Lung Cell Atlas. These atlases are published and thus have gone through validation in the form of peer-review. However, for new scvi-hub atlases that may be uploaded to scvi-hub in the future, we will include only the models with either a pre-print/publication or model evaluation results in the model card in scvi-hub, as described here. Users who would like to map their data to an scvi-hub atlas that does not meet these requirements can upload the desired atlas and run ArchMap's benchmarking process as described here. We have also added icons to all HCA approved and published atlases in the ArchMap website to distinguish these atlases from others.

To communicate this in the paper we have added the following text to the methods section:

> "Publicly available scvi-hub atlases that are compatible with scArches mapping include all compartments of the multiple-organ Tabula Sapiens atlas and the Human Lung Cell Atlas. These atlases are published and thus have gone through validation in the form of peer-review. However, for atlases uploaded to scvi-hub in the future, we will make available on Archmap only the atlases with a pre-print, publication, or model evaluation results in the model card in scvi-hub, as described here. Users who would like to map their data to an scvi-hub atlas that does not meet these requirements can upload the desired atlas and run ArchMap's benchmarking process as described here."

25. Presence score: My understanding is that it measures which reference cell states are present in the query dataset. Could the authors clarify this and rephrase lines 141-143.

The explanation of the presence score in the main text has been rephrased for clarity. You are correct in saying it measures which reference cell states are present in the query dataset. We have changed the text to the following:

> "Additionally, a built-in presence score measures which reference cell states are present in the query dataset, which can be used to quantify the suitability of a reference for a particular query dataset (see Methods)."

We have included a detailed explanation on how the presence score is calculated in the Methods section:

> "The presence score quantifies reference cell state representation in the query dataset, based on shared neighborhood structure between reference and query cells. Using a weighted k-nearest neighbor (WKNN) graph derived from the embedding space, we count how frequently each reference cell is identified among the k nearest neighbors (k = 15) of query cells. This results in an initial presence score p for each reference cell, defined as
>
> $$p_i = \sum_{q \in Q} w(q,i)$$
>
> where $w(q,i)$ is the WKNN weight of reference cell i in the neighborhood of query cell q.
>
> To account for similarity structure within the reference dataset, the initial presence score for each reference cell is diffused using a random walk with restart performed over the reference cell transition probability matrix T, constructed from a reference KNN graph (k = 15):
>
> $$p^{(t+1)} = (1-\alpha) T p^{(t)} + \alpha p^{(0)},$$
>
> with restart probability $\alpha = 0.1$. The process is iterated for 100 rounds.
>
> Presence scores are then log-transformed, clipped to the 1st and 99th percentiles, and normalized to $[0, 1]$."

26. When discussing minification, the authors should cite scvi-hub to demonstrate why this is a reasonable step.

We have cited the scvi-hub publication when discussing minification in the methods section.

27. Line 277: In the GitHub API the developers use load_query_anndata of scVI and scANVI.

We have edited the text to clarify that the load_query_data method is from the scVI and scANVI codebases.

28. Line 300: It is unclear to me to which Cell Number Requirement the authors are referring? Cellxgene is compatible with millions of cells.

We are indeed referring to the cell number requirements of cellxgene (4.6 million cells). The subsetting of the reference is done to keep to these limits. This is necessary for example for the PBMC atlas, which has over 7 million cells. The reason that we subset all atlases, even if they conform to cellxgene cell number requirements is to allow for increased mapping and post-processing speed even for smaller atlases. The subsetting however is only done for the visualization and only the reference is subsetted, not the query. The subsetting is done based on cell type proportions, such that cell type proportions are preserved in the visualization. We have added the following text in the paper to explain this:

> “Before a CellxGene instance is created for visualisation of the mapping results, the reference cells of the joint representation are subsetted to speed up computation of the UMAP and to guarantee conformity to CellxGene cell number requirements for the case of a large reference such as the PBMC atlas which has 7 million cells. For all atlases, the subsetting is done in a way that preserves the cell type proportions in the reference data and keeps the ratio between reference and query no smaller than 5:1.”

(Remarks on code availability):

No issues with the published code. Navigation is easy and I can follow aspects of the package. See above for detailed questions about the code.

Response to reviewer 3

Here, Lotfollahi et al present ArchMap which is a webplatform for mapping scRNAseq profiles to a reference dataset. The website is based on their previously published scArches method, and an key feature for the website is the inclusion of annotated reference atlases. Importantly, the website is modular and it allows users to upload new atlases, to reduce the chances that it will become outdated in the near future.

Given the number of atlas datasets that are currently being generated, reference mapping is an increasingly important application. ArchMap is a potentially valuable addition as it could

simplify the process for readers without bioinformatics expertise. Hence, I applaud the idea, but the implementation leaves much to be desired, and I believe that the following major issues must be resolved:

1. Please discuss how ArchMap compares LungMAP (https://www.nature.com/articles/s41467-023-40173-5). This seems relevant given the focus on lung data, so it would be useful to see a comparison between the two.

Thanks for raising this need for clarification! LungMAP is a consortium that generates data and allows you to search through their datasets on an online portal. LungMap does not perform any query to reference mapping and thus does not support users to analyse new datasets. These aims are different from ArchMap, which is an online tool to map new datasets to existing reference atlases. The reviewer highlights the LungMAP CellRef reference atlas paper. This is similar to the HLCA, except that it uses a guided reference construction method that is not compatible with scArches and thus cannot be used with ArchMap. While ArchMap includes an HLCA reference, it is actually not lung focused, but instead includes 18 core reference atlases, of which 4 are lung-related and 14 are not. It also includes 21 Tabula Sapiens atlases from scvi-hub, of which only 1 is lung. With the active development of the human lung cell atlas V2, we will be able to provide a larger integrated lung atlas for reference mapping that incorporates many LungMAP samples.

2. Can users adjust the threshold for quantification metrics, such as the uncertainty score, to identify novel cells in query dataset? And how was the 0.5 threshold determined in the manuscript for uncertainty score?

We have set the threshold for cell uncertainty scores to 0.5 for labelling query cells as unknown as an uncertainty above 0.5 is more uncertain than certain for any cell type label and no label stands out as a strong prediction. Furthermore, at higher uncertainties it is possible that multiple labels are equally likely. In the case users are interested in considering novel cells in the query dataset that may have high uncertainty, we have also included in the output a column with all transferred labels for each query cell without any threshold used. We named this column ”{cell_type_key}_prediction_{classifer_name}”, where cell_type_key is the key in the obs attribute of the cell type level that was mapped to and classifer_name is the name of the chosen classifier (e.g. knn)). This allows users to also obtain transferred labels for query cells with uncertainty greater than 0.5. Furthermore, the ”{cell_type_key}_uncertainty_euclidean” column can be used to view the exact uncertainty scores for all query cells. Thus, users can adapt the uncertainty manually in CellxGene using the available columns mentioned above and there is no need to do this before mapping. We have made this clear in the main text by commenting that the user has access to transferred labels and uncertainty scores for all cells and the cut-off of 0.5 is only a suggestion:

> “Users can relate the uncertainty scores with the transferred labels manually in CellxGene for all cells. A suggested cut-off of 0.5 is given in ArchMap for the uncertainty scores, where values higher than this are recommended to be labelled as unknown as the resulting label is more uncertain than certain (Methods).”

3. In some models the features are not changeable. Ideally, they should include both ensemble ID and gene names. I realize that this may depend on how the reference was processed, but I believe that it is something that will annoy many users.

Indeed this is related to how the specific references were built. However, we agree with the reviewer that this may annoy users. Thus, we now do automatic feature conversion between gene symbols and Ensembl IDs in the mapping pipeline. We have obtained Biomart mapping tables for the conversions of both mouse and human following this tutorial. In order to give the user the opportunity to improve on this conversion in case of insufficient performance, we let the user know that this conversion was done internally, as shown in the screenshot in response 12 for Reviewer 2 (the warning is in green). We have also added this explanation to the methods section.

4. I tried to use the website, not just myself but several people in the lab. All of them encountered various errors that prevented them from evaluating the quality of the results. For example, when uploading a pancreatic cancer dataset to map to the pancreas, the following
Happened:

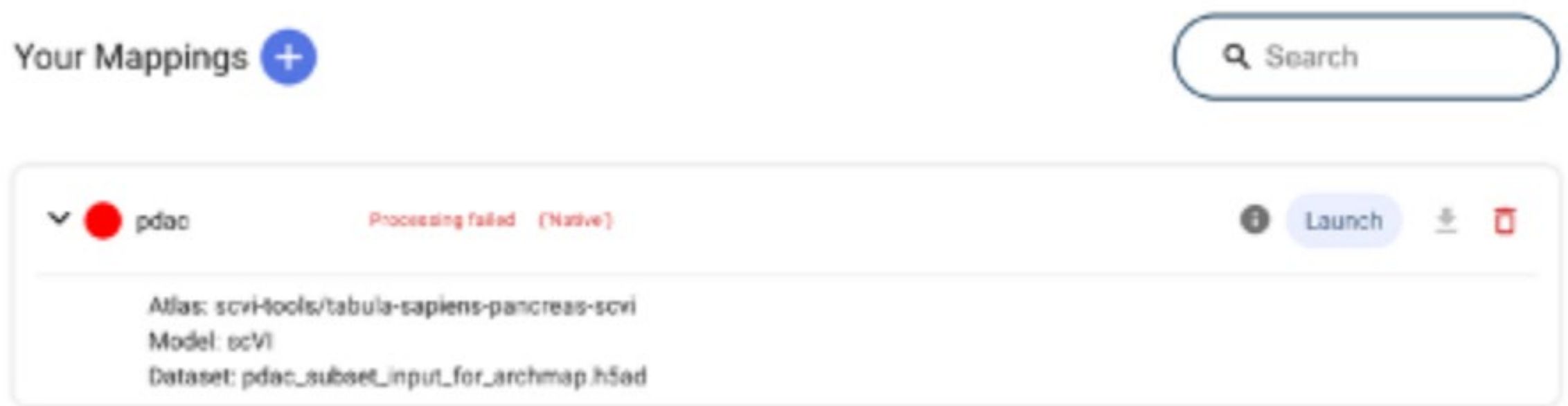

This error was to do specifically with the scvi-hub atlases, very sorry about that. This has been addressed and is now resolved. To stress test ArchMap we have also engaged 3 different labs to test ArchMap across references and query datasets. We have also provided a sheet that mentions a subset of query datasets that have been successfully tested in ArchMap by the authors for all core and scvi-hub reference atlases. We believe this can be used as a suitable reference to show the extent of the testing of ArchMap's mapping tool. This sheet also includes datasets that were converted from rds format to h5ad using this tutorial. We have also added improved error reporting. This includes specific error messages for h5ad formatting issues or missing data such as zero genes or cells, missing var or obs names, missing X attribute. For unforeseen formatting issues, we have added a generic error message that encourages the user to send an email to the ArchMap team with the specified email address with the error message and their mapping name so that we can troubleshoot the error with them. Additionally, we have implemented automatic error logging in the ArchMap database for all user mappings to be able to fix any issues encountered by users when mapping their data in ArchMap. This allows us to fix any errors without the user needing to inform us through GitHub issues or through the website contact form. We have been actively using this method for the last five months and, as a result, we have received no new errors for all user mappings (a total of 28) from 18.10.2025 to 19.11.2025 (the time

of writing this) according to our database. We would like to note that these mappings in this time period are all from external users (not including researchers we have recruited for beta testing), showing the increased robustness and usability of the website.

Would be more helpful to use more descriptive phrases like “loading model,” “preparing data,”
“fine-tuning,” or “mapping” to make it clear which part of the project is currently running.

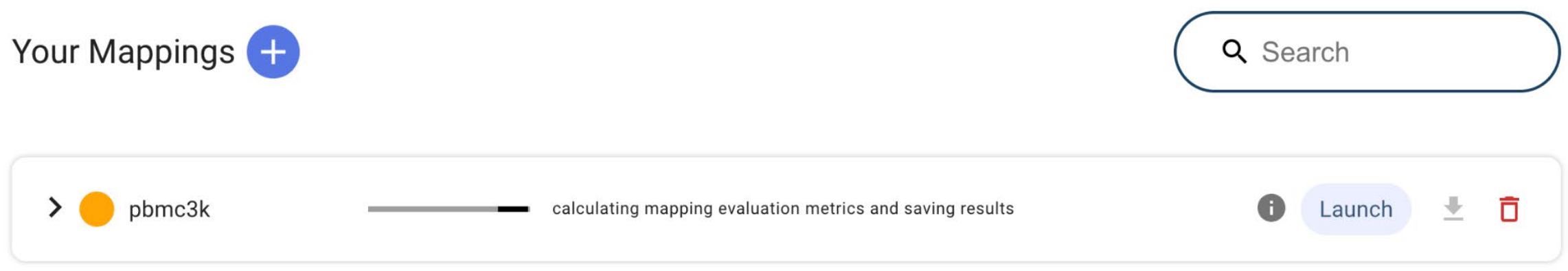

Thank you for this suggestion. We have now added descriptive phrases during the mapping (see example above) including "fetching and preprocessing data", "mapping query", "calculating mapping uncertainty scores", "transferring labels from reference to query", "concatenating reference and query results", and "calculating mapping evaluation metrics and saving results".

Minor:
In Figure 1d (iii), the distribution shows that the purple cells represent those with high uncertainty. However, in the explanation of Figure 1d (iv), it appears that the orange cells are indicated as the ones with high uncertainty based on the description “Marker gene expression patterns of CTHRC1 and SERPINF1 show higher expression in high and low uncertainty cells,
respectively.” Please double-check the labels or descriptions.

The distribution had been wrongly coloured. We have updated the distribution to the correct colour code where orange represents high uncertainty. We apologize for this oversight and thank the reviewer for pointing this out.

Only some models are available for all reference. Why the other models are missing?

Not all models are available for all atlases. This is because we have not done any training on reference data ourselves. All atlases’ models are taken from existing publications or pre-prints. For most of these atlases only a single model was used by the atlas builders for integration. Since we did not want to interfere with original authors’ choices on the embedded atlases (a lot of validation work typically went into each of these reference atlases), we did not redo any integrations ourselves. To clarify this in the main text of the manuscript, we have included the following:

> “All reference atlases available in ArchMap are the extensively evaluated, published and, where available, consortium-approved versions (e.g., by the Human Cell Atlas[27]; HCA) of the atlases. We do not retrain models ourselves, as this voids the extensive

performance evaluation and community based annotation that is typically done for HCA atlases.”

“Ensure are stored in the var_names AnnData object attribute of your query.” marked for scVI
Hub Atlases. What feature should users use here?

Since we now do automatic feature conversion between gene symbols and Ensembl IDs in the mapping pipeline, this requirement has been removed from the website.

The demo data is not available when we tried to use it

All demo datasets have now been added for each atlas. We apologize for this oversight. Citations for all demo datasets can be found in the ArchMap docs

Editor’s comments:

Dear Dr. Theis,

Thank you for submitting your revised manuscript "ArchMap: A web-based platform for reference-based analysis of single-cell datasets" (NG-BC66689R). It has now been seen by the original referees and their comments are below. The reviewers find that the paper has improved in revision, and therefore we'll be happy in principle to publish it in Nature Genetics, pending minor revisions to satisfy the referees' final requests and to comply with our editorial and formatting guidelines.

If the current version of your manuscript is in a PDF format, please email us a copy of the file in an editable format (Microsoft Word or LaTex)-- we can not proceed with PDFs at this stage.

We are now performing detailed checks on your paper and will send you a checklist detailing our editorial and formatting requirements soon. Please do not upload the final materials and make any revisions until you receive this additional information from us.

Thank you again for your interest in Nature Genetics Please do not hesitate to contact me if you have any questions.

Sincerely,

Margot Brandt, PhD
Senior Editor
Nature Genetics

Dear Margot,

Thank you very much for the opportunity to publish our work in Nature Genetics. We have carefully gone through the referee’s final requests and have made the corresponding revisions.

Specifically, we have (i) added the requested features to the ArchmMap platform, including the ability to download transferred cell type annotations as a separate file and to preview output file size prior to download; (ii) refined the ArchMap tutorial notebook and implemented an automated testing script to ensure continued functionality, confirming that all notebooks run successfully; and (iii) included a clarification in the main text regarding the limitations of the uncertainty metric for identifying novel or disease-associated cell states.

In addition, we have reviewed the editorial and formatting checklist provided by the journal and ensured that the manuscript, figures, and supplementary materials fully comply with all requirements.

We thank you and the reviewers for the thoughtful feedback, which has helped us improve the manuscript.

Best regards,
Fabian, Malte, Mo, on behalf of all authors

In the following, reviewers' comments appear in *blue*, our point-by-point answers appear in **black**, and passages altered in the manuscript are written inside quotation marks and *italicized* in the response letter.

Response to Reviewer 1

Reviewer #1 (Remarks to the Author):

In the revised submission, the authors have nicely addressed my original comments. I was able to test the webtool and it worked as advertised. I only have a few remaining comments/suggestions on this front:

1. I think it would be useful to have an idea of the size of the tar.gz file that will be downloaded before starting the download. In addition, I could imagine that some users would appreciate being able to download a simple text file with just the query cells and the annotations produced by the tool (which would be much smaller than the current tar.gz file, and also easier to integrate with existing workflows and processed data objects).

Thank you very much for this valuable feedback and suggestion! We have now implemented these features. In particular, after mapping, the user can see how large the tar.gz file is that contains the model.pt file and h5ad file containing the atlas and query data and label predictions. We have also added a separate text file, as you have suggested, that contains the predicted cell type labels for each cell at the different available cell type granularities of the chosen atlas. A screenshot of how these features are implemented in ArchMap is shown below.

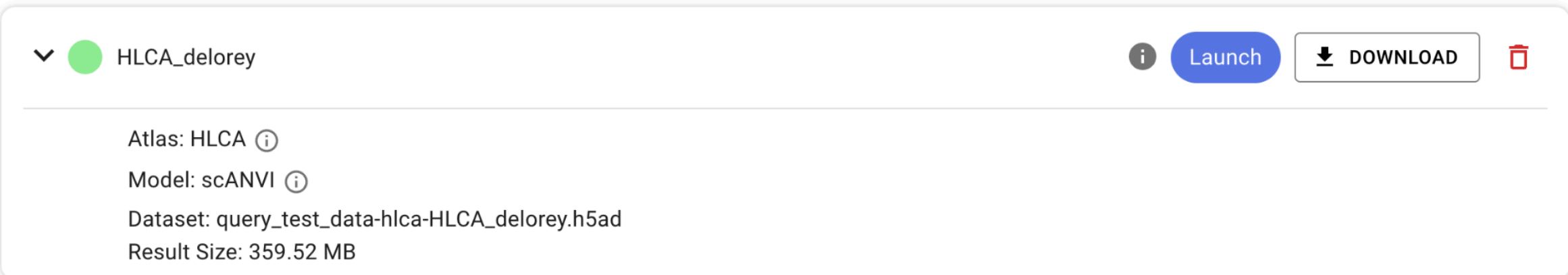

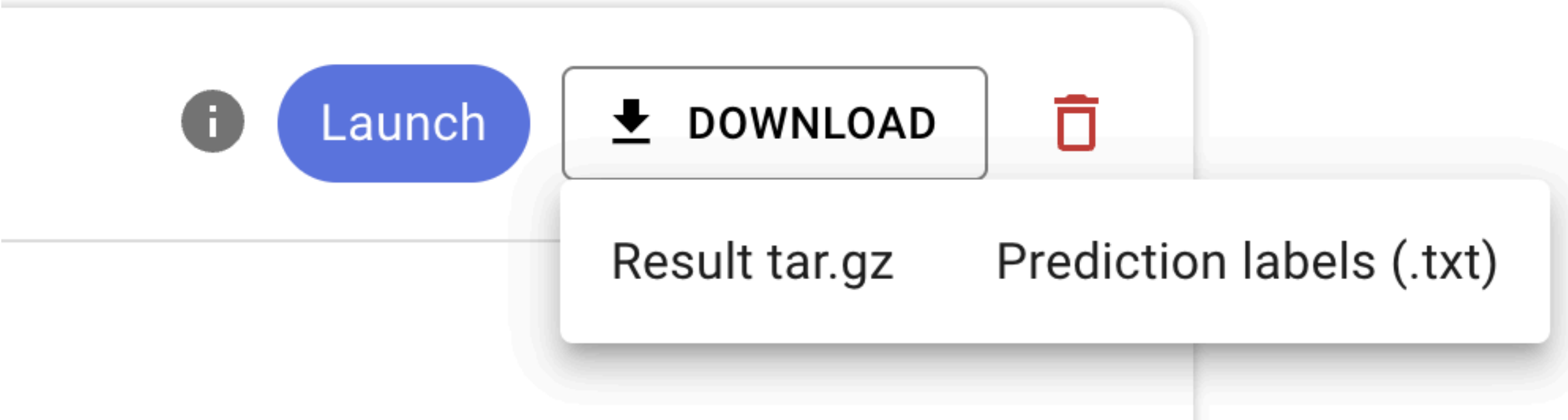

2. What is the difference between reference datasets with Modalities "RNA-seq" and "rna"?

We apologize for this oversight in the modality reference of the atlases and thank you for pointing this out to us. Indeed, both these terms refer to the same modality: scRNA-sequencing. We have made sure that all atlases (and the default modality for all future uploaded atlases) are labelled as “RNA-seq”.

3. The code in the 'CheckFormatsRds.ipynb' notebook does not execute properly (it fails at Bioconductor installation) since the latest colab runtime version provides R v4.5.2, while Bioconductor version 3.20 (which is hardcoded in the notebook) requires R v4.4. I would suggest to decide on a strategy here - either fix the R version to an old one (which may work for conversion as long as the AnnData format does not change) or don't fix the Bioconductor version (and make sure that the code still runs properly with newer Bioconductor releases). In general (see also the code comments below), this may point to a broader issue - how do the authors ensure that code distributed via notebooks is regularly tested and kept up-to-date and functional?

Thank you for bringing this problem up to us! We agree on the importance of regular testing of notebooks to keep them functional. First, to fix the issue with the 'CheckFormatsRds.ipynb' notebook, we have removed the hardcoding of the Bioconducter version as suggested. Second, we have put in place a testing script for all notebooks which runs each notebook through fully and exits when an error is encountered. This script can then be run on a schedule to maintain functionality and has been added to our internal on-boarding documentation that can be accessed by new maintainers of ArchMap

Reviewer #1 (Remarks on code availability):

1. I attempted to follow the instructions from https://archmap-docu.readthedocs.io/en/latest/tutorials/map_local.html, including downloading the example data from the Google Drive and running the tutorial from

https://github.com/theislab/archmap_data/blob/mappingjob/mapping/tutorial.ipynb, but it failed with the following error:
>>> from scarches_api.utils.utils import replace_X_on_disk, check_h5ad_format
Traceback (most recent call last):
File "<stdin>", line 1, in <module>
ImportError: cannot import name 'check_h5ad_format' from 'scarches_api.utils.utils' (archmap_data/mapping/scarches_api/utils/utils.py)
I have scarches version 0.6.1

We apologize for not detecting this error beforehand! We updated the naming of a utils function previously and this update was not made in the tutorial notebook. As mentioned above, we have now set up a testing script for all notebooks that can be run upon updating the code in the ArchMap repo to make sure that the notebooks are still functional after changes are made. We believe this will work well to circumvent future runtime errors in the notebooks and will help to keep all notebooks up-to-date.

2. The code in the tutorial could be cleaned up - for example, some modules (scanpy, pandas) are imported several times

Thank you very much for bringing this up to us! We have now cleaned up the tutorial by removing any redundant imports and have tested the notebook to confirm that it is fully functional.

Response to Reviewer 2

Reviewer #2 (Remarks to the Author):

The reviewers have significantly improved the manuscript as well as the web portal. In my opinion this article now mertis publication in Nature Genetics. Especially the benchmarks provided for training speed (number of epochs) as well as missing genes will benefit the community beyond ArchMap.
I would like the authors to address that the provided distance metric for detection of new cell-types might not be robust in all scenarios and that manual verification remains key after automatic cell-type transfer. For example, if a new cell-type is relatively close to an existing cell-type such as monocytes compared to macrophages, these cells would not be flagged but the annotation is still inaccurate.

We thank the reviewer for the time they have devoted to evaluating our revisions. We agree that it is important to clarify in the paper the limitations of the uncertainty distance metrics in identifying novel or disease-associated cell states. Thus, we have included the following statement in the main text:

“Using uncertainty scores alone to identify novel or disease-associated cell states may not always be reliable. Residual batch effects or low-quality cells (e.g., low gene counts or high mitochondrial content) can also yield high uncertainty and lead to misinterpretation. Therefore, high-uncertainty cells should be manually validated after label transfer, for example using differential expression analysis as shown above.”

Response to Reviewer 3

The authors have addressed all of my concerns, and the website worked very nicely in our tests.

We sincerely thank the reviewer for their evaluations that have helped to improve the ArchMap website and our paper submission.

Here, Lotfollahi et al present ArchMap which is a webplatform for mapping scRNAseq profiles to a reference dataset. The website is based on their previously published scArches method, and an key feature for the website is the inclusion of annotated reference atlases. Importantly, the website is modular and it allows users to upload new atlases, to reduce the chances that it will become outdated in the near future.

Given the number of atlas datasets that are currently being generated, reference mapping is an increasingly important application. ArchMap is a potentially valuable addition as it could simplify the process for readers without bioinformatics expertise. Hence, I applaud the idea, but the implementation leaves much to be desired, and I believe that the following major issues must be resolved:

1. Please discuss how ArchMap compares LungMAP (https://www.nature.com/articles/s41467-023-40173-5). This seems relevant given the focus on lung data, so it would be useful to see a comparison between the two.

2. Can users adjust the threshold for quantification metrics, such as the uncertainty score, to identify novel cells in query dataset? And how was the 0.5 threshold determined in the manuscript for uncertainty score?

3. In some models the features are not changeable. Ideally, they should include both ensemble ID and gene names. I realize that this may depend on how the reference was processed, but I believe that it is something that will annoy many users.

4. I tried to use the website, not just myself but several people in the lab. All of them encountered various errors that prevented them from evaluating the quality of the results. For example, when uploading a pancreatic cancer dataset to map to the pancreas, the following happened:

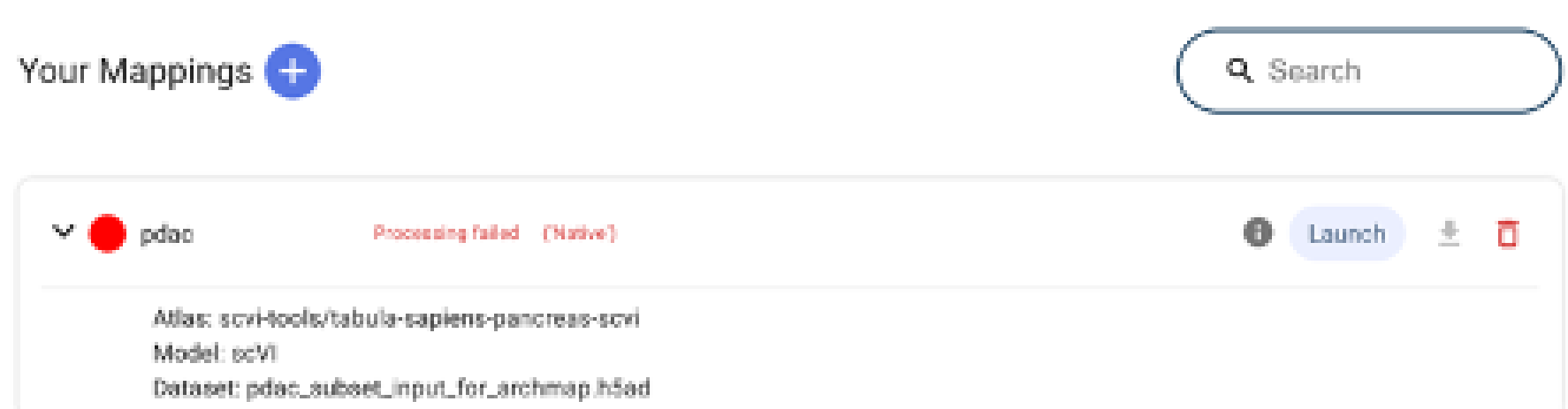

Would be more helpful to use more descriptive phrases like “loading model,” “preparing data,” “fine-tuning,” or “mapping” to make it clear which part of the project is currently running.

Minor:

In Figure 1d (iii), the distribution shows that the purple cells represent those with high uncertainty. However, in the explanation of Figure 1d (iv), it appears that the orange cells are indicated as the ones with high uncertainty based on the description “Marker gene expression

patterns of CTHRC1 and SERPINF1 show higher expression in high and low uncertainty cells, respectively.” Please double-check the labels or descriptions.

Only some models are available for all reference. Why the other models are missing?

“Ensure are stored in the var_names AnnData object attribute of your query.” marked for scVI Hub Atlases. What feature should users use here?

The demo data is not available when we tried to use it
